# LYRM2 Promotes the Growth and Metastasis of Hepatocellular Carcinoma via Enhancing HIF‐1α‐Dependent Glucose Metabolic Reprogramming

**DOI:** 10.1111/jcmm.70241

**Published:** 2024-12-11

**Authors:** Bingfu Fan, Yueqin Zhang, Lu Zhou, Zhongchun Xie, Jie Liu, Chengwu Zhang, Changwei Dou

**Affiliations:** ^1^ General Surgery, Cancer Center, Department of Hepatobiliary & Pancreatic Surgery and Minimally Invasive Surgery Zhejiang Provincial People's Hospital, Affiliated People's Hospital, Hangzhou Medical College Hangzhou China; ^2^ Graduate School of Bengbu Medical College Bengbu China

**Keywords:** glycolysis, hepatocellular carcinoma, HIF‐1α, LYRM2, tumour growth and metastasis

## Abstract

Hepatocellular carcinoma (HCC) is a foetal malignancy with dismal overall survival. The molecular mechanism underlying the progression of HCC remain largely unknown. LYR motif containing 2 (LYRM2) has been identified as an oncogene in colorectal cancer; however, its expression, functions and molecular mechanism in the context of HCC has not been investigated. Data derived from The Cancer Gemome Atlas, along with findings from our patients' cohort, indicate that LYRM2 expression is elevated in HCC tissues and correlates with adverse clinicopathological features and prognosis in HCC patients. Subsequent research into the biological functions of LYRM2 has revealed that it promotes the proliferation, migration, invasion and epithelial‐mesenchymal transition of HCC cells, both in vitro and in vivo. Mechanistic insights have shown that LYRM2 interacts with HIF‐1α, enhancing the protein stability of HIF‐1α, which in turn increases cellular glycolysis and inhibits mitochondrial respiration. Moreover, the glucose metabolic reprogramming mediated by LYRM2 is implicated in its role in promoting HCC growth and metastasis. Collectively, this study identifies that LYRM2 as a novel oncogenic protein in HCC and elucidates its contribution to HCC progression through enhancing HIF‐1α‐dependent glucose metabolic reprogramming.

## 1 Introduction

Hepatocellular carcinoma (HCC) is a highly fatal disease that is frequently diagnosed at an advanced stage. It ranks as the seventh most prevalent cancer worldwide, with 900,000 newly diagnosed cases and 800,000 deaths reported annually [1]. Despite significant progress in the treatment of HCC, the long‐term survival of HCC patients remains dismal. The poor prognosis is primarily attributed to the rapid growth, the occurrence of intra‐hepatic or distant metastasis, and postoperative recurrence [2]. Consequently, there is an urgent need to explore the underlying mechanisms of HCC progression, as this may facilitate the identification of novel therapeutic targets for HCC patients.

The Warburg effect is a hallmark of tumour cells, which exhibit increased glucose uptake and lactate production even in oxygen‐rich microenvironment [3]. This phenomenon, characterised by increased aerobic glycolysis, enables cancer cells to acquire more energy and intermediate metabolites necessary for the biosynthesis of different biological processes [4]. Accumulating researches indicate that mitochondrial dysfunction is an important reason for the augmented aerobic glycolysis in cancer cells [5, 6]. The aberrant activation of oncogenes, such as c‐Myc and HIF‐1α, as well as the inactivation of tumour suppressors including p53, contributes to the reprogrammed glucose metabolism and the mitochondrial dysfunction [7, 8, 9, 10]. Furthermore, elevated expression and activity of key metabolic genes, including hexokinase 2 (HK2), pyruvate kinase muscle 2 (PKM2) and lactate dehydrogenase A (LDHA), have been documented in various human cancers and are correlated with poorer patient survival outcomes [11, 12].

LYR motif containing 2 (LYRM2) is a conserved member of the leucine‐tyrosine‐arginine motif‐containing proteins (LYRMs), which regulates mitochondrial activities by serving as a subunit of mitochondrial complexes. Aberrant expression or dysfunction of LYRMs has been reported in various human cancers, including oesophageal carcinoma [13], breast cancer [14] and lung cancer [15]. Currently, the understanding of LYRM2, particularly regarding its role in human cancers, is limited. In colorectal cancer, LYRM2 has been shown to promote the oxidative phosphorylation and the growth of cancer cells through its interaction with mitochondrial complex I [16]. However, the role of LYRM2 in HCC remains unexplored. This study represents the first investigation into the role of LYRM2 in HCC, focusing on its expression patterns, prognostic significance, functional influence and underlying molecular mechanism. These findings demonstrate that LYRM2 is overexpressed in HCC and promotes the growth and metastasis of HCC cells both in vitro and in vivo. Furthermore, LYRM2 exerts its oncogenic effects in HCC by enhancing HIF‐1α‐dependent glucose metabolic reprogramming.

## 2 Materials and Methods

### 2.1 Clinical Tissues and Data Collection

Sixty‐three paired HCC tissues (T) and adjacent non‐tumour tissues (NT) were collected from patients who underwent curative operations for HCC at the Zhejiang Provincial People's Hospital (Hangzhou, China) between January 2020 and August 2022. Clinical data were extracted from the medical records of the enrolled patients. All procedures were approved by the Ethics Committee of the Zhejiang Provincial People's Hospital and adhered to the principles outlined in the Helsinki Declaration of 1964 and later versions.

### 2.2 Data Mining of Online Databases

The UALCAN platform (https://ualcan.path.uab.edu/index.html) and GEPIA platform (http://gepia.cancer‐pku.cn/index.html) were utilised to investigate the expression levels of LYRM2 in HCC tissues, as well as to assess the relationship between LYRM2 level and patients' clinicopathological features and survival, based on the data from TCGA database. The correlation between LYRM2 expression and genes, including PKM2, LDHA, HK2, GLUT1 and HIF‐1α, was examined using GEPIA platform (http://gepia.cancer‐pku.cn/index.html). The R2 platform (https://hgserver1.amc.nl/cgi‐bin/r2/main.cgi?open_page=login) was employed to evaluate the expression levels of LYRM2 in GEO datasets (GSE45436 and GSE6764).

### 2.3 Cell Culture and Transfection

Immortalised normal liver cell MIHA and HCC cell Hep3B were purchased from the Institute of Biochemistry and Cell Biology, Chinese Academy of Science (Shanghai, China). HCCLM3, MHCC97L and MHCC97H were obtained from the Liver Cancer Institute of Fudan University. These cell lines were cultivated in Dulbecco's modified Eagle's medium (DMEM) (Hyclone) with foetal bovine serum (10%, Gibco, Thermo Fisher Scientific Inc.), penicillin (100 IU/mL, Gibco) and streptomycin (100 μg/mL, Gibco) at a temperature of 37°C in a humid atmosphere containing 5% CO_2_.

To knockdown LYRM2 or HIF‐1α in HCC cells (HCCLM3, MHCC97H or Hep3B), a small hairpin RNA (shRNA) expression vector was constructed using the pSilencer 2.1‐U6 puro vector (Thermo Fisher Scientific). The sequences of shRNAs targeting LYRM2 and HIF‐1α were as follows: shLYRM2‐#1, CCGGGTCACTAGAATGGCTGTAATTACTCGAGTAATTACAGCCATTCTAGTGATTTTTG, shLYRM2‐2, CCGGGATGATTACTCAAGGCAATATCTCGAGATATTGCCTTGAGTAATCATCTTTTTG, sh‐HIF1α, TGCTCTTTGTGGTTGGATCTA, sh‐NC, TTCTCCGAACGTGTCACGT. To construct LYRM2 overexpression vector, LYRM2 cDNA was amplified and subsequently inserted into the pCDNA‐3.1 vector (Invitrogen, V790‐20), with an empty vector (EV) serving as the control group. The transfection of the aforementioned shRNAs or plasmids was performed with Lipofectamine 2000 reagent (Invitrogen; Thermo Fisher Scientific Inc.). Cell lines exhibiting stable overexpression or knockdown of LYRM2 were established through transient transfection followed by selection with G418 (500 μg/mL). Fourty‐eight hours post‐transfection, the efficacy of the overexpression or knockdown of the target genes (LYRM2 or HIF‐1α) was confirmed using quantitative real‐time polymerase chain reaction (qRT‐PCR) or western blot.

### 2.4 Quantitative Real‐Time Polymerase Chain Reaction (qRT‐PCR)

Total RNA was extracted from human tissue samples or cultured cells using TRIzol reagent (Invitrogen). cDNA was synthesised from the extracted RNA using the PrimeScript cDNA Synthesis Kit. SYBR Green reagents (Takara, Japan) were used for qRT‐PCR, with the primer sequences listed in Table S1. The qRT‐PCR reactions were performed using the ABI 7500 fast PCR System with 100 ng of cDNA. The thermocycling conditions were described as follows: an initial denaturation at 95°C for 60 s, followed by 40 cycles consisting of 10 s at 95°C and 30 s at the annealing temperature of 95°C. The relative mRNA levels were normalised to β‐actin, which was used as an internal control in this study and were calculated using the 2^−ΔΔCt^ method.

### 2.5 Western Blot

For protein extraction, cultured cells or tissue specimens were treated with radioimmunoprecipitation assay (RIPA) lysis buffer (Beyotime Institute of Biotechnology), which was supplemented with a protease inhibitor cocktail (Roche). The supernatant, containing cellular or tissues' protein, was subjected to protein concentration measurement using a bicinchoninic acid (BCA) protein assay kit (Thermo Fisher Scientific). Protein samples (25–30 μg per lane) were mixed with loader buffer, subjected to separation on SDS‐PAGE (BioRad, Berkeley) and then transferred to polyvinylidene fluoride (PVDF) membranes (BioRad). Following a blocking step with 5% non‐fat dry milk, the membranes were incubated with primary antibodies at 4°C for 12 h. The following antibodies used in this study included: anti‐LYRM2 (1:500, ab106686, Abcam, USA), anti‐E‐cadherin (1:1500, A11492, Abclonal, Wuhan, China), anti‐N‐cadherin (1:1000, A19083, Abclonal), anti‐vimentin (1:2000, A19607, Abclonal), anti‐HIF‐1α (1:500, ab1, Abcam), anti‐c‐Myc (1:2000, A1309, Abclonal), anti‐p53 (1:1000, A0263, Abclonal), anti‐LDHA (1:1000, A0861, Abclonal), anti‐HK2 (1:1500, A20829, Abclonal), anti‐GLUT1 (1:1000, A6982, Abclonal), anti‐PKM2 (1:1000, A20991, Abclonal), anti‐AKT (1:3000, A18120, Abclonal), anti‐ERK (1:1500, A16686, Abclonal), anti‐JNK (1:1000, A18287, Abclonal), anti‐p‐AKT (1:1500, AP0140, Abclonal), anti‐p‐ERK (1:500, AP0974, Abclonal), anti‐p‐JNK (1:500, AP1337, Abclonal), anti‐HIF‐2α (1:1000, Ab207607, Abcam) and anti‐β‐actin (1:10,000, AC004, Abclonal). Then, the membranes were blotted with secondary HRP‐conjugated antibodies (1:5000, Abcam)at room temperature for 1 h. The levels of the intended proteins were detected using Pierce ECL Plus Substrate (Thermo Fisher Scientific).

### 2.6 Cell Proliferation Assay

The relative cellular proliferation ability was evaluated using the Cell Counting Kit‐8 (CCK‐8; Dojindo, Kumamoto, Japan). Cells were prepared at a density of 3 × 10^3^ cells/100 μL and subsequently seeded into 96‐well plates (*n* = 6 for each time point). Following culture periods of 0, 24, 48 or 72 h, the absorbance at 450 nm was measured using a spectrophotometer after the addition of 10 μL of CCK‐8 to each well, followed by a 2‐h incubation at room temperature.

### 2.7 Colony Formation Assay

To measure the colony formation ability, HCC cells exhibiting either overexpression or knockdown of LYRM2 were seeded into 6‐well plates at a density of 1 × 10^3^ cells per well and cultured for a duration of 2 weeks. Subsequently, the cell colonies were fixed and stained with 1% crystal violet (Beijing Solarbio Science & Technology) for 5 min at room temperature. The number of cell colonies was counted using a light microscope.

### 2.8 Transwell Assay

Transwell migration and invasion assays were employed to assess the metastatic potential of HCC cells. To evaluate invasive ability, the membrane of an 8 μm Transwell insert was coated with Matrigel (BD Biosciences) at room temperature for 2 h. DMEM medium supplemented with 20% FBS was introduced into the bottom chamber. Two hundred microlitres serum‐free medium with 5 × 10^4^ HCC cells was added into upper chamber, while 600 μL of medium containing 20% FBS was added into the lower chamber. Following a 24‐h incubation, cells that had migrated through a membrane or invaded across the Matrigel were fixed with methanol for 20 min and subsequently stained with 1% crystal violet for 5 min at room temperature. The number of migrated or invaded cells was quantified using a microscope.

### 2.9 Xenograft Tumour Growth Assay and In Vivo Metastasis Assay

Animal study procedures were approved by the Institutional Animal Care and Use Committee of the Zhejiang Provincial People's Hospital (Hangzhou, China). All mice (BALB/c nu/nu, female, weight range, 17–20 g, age, 5 weeks) were purchased from Shanghai Slac Laboratory Animal Co. The mice were maintained in a pathogen‐free environment, with a temperature of 20°C, humidity at 50% and a light–dark cycle of 12 h. For the xenograft tumour growth assay, 5 × 10^6^ stably LYRM2‐silencing or negative control HCCLM3 cells were suspended in 100 μL of phosphate‐buffered saline (PBS) and subcutaneously inoculated into the left flank of nude mice (*n* = 5 per group). Tumour volume was measured for every 5 days using the calliper over a period of 25 days. Following the after sacrifice of the mice via CO_2_ asphyxiation, the subcutaneous tumours were harvested for immunohistochemical (IHC) staining. The volume of tumour nodules (*V*) was calculated using the formula of *V* = (*a* × *b* ^2^)/2 where ‘*a*’ represents the largest tumour diameter, and ‘*b*’ represents the smallest tumour diameter. In the tail vein metastasis model, 1 × 10^6^ HCCLM3 cells from the control group or those with stable LYRM2 knockdown were injected into the tail vein of nude mice (*n* = 5 per group). Six weeks after the tail vein injection, the lung tissues were collected and subjected to haematoxylin and eosin (H&E) staining to identify the lung metastases. In vivo assays were approved by the Institutional Animal Care and Use Committee of the Zhejiang Provincial People's Hospital (Hangzhou, China).

### 2.10 IHC Staining

For IHC staining, tissue sections from patients or nude mice underwent a series of procedures, including deparaffinisation and rehydration. The sections were then incubated in citrate buffer (pH 6.0, 0.01 M) for antigen retrieval using a microwave oven at 95°C for 10 min, followed by cooling to room temperature. Sections were treated with H_2_O_2_ for 10 min to block endogenous peroxidase activity. The following primary antibodies were employed to assess the expression levels of specific proteins in the tissue sections: LYRM2 (1:100, ab106686), HIF‐1α (1:50, ab1), Ki67 (1:200, ab15580), E‐cadherin (1:100, A11492), Vimentin (1:100, A19607), N‐cadherin (1:200, A19083), p53 (1:100, A0263), c‐Myc (1:200, A1309) and HIF‐2α (1:50, Ab207607). The IHC staining results were evaluated based on the intensity and percentage of positive staining.

### 2.11 Glucose Uptake and Lactate Production Detection Assays

For glucose uptake and lactate production assays, culture media from HCC cells subjected to the specified treatments were collected. The concentrations of glucose and lactate in the culture media were measured using a glucose colorimetric assay kit (BioVision, SF, USA) and a lactate assay kit (Sigma, MO, USA), respectively. The concentration of cellular protein was utilised for the normalisation of glucose and lactate concentration.

### 2.12 Oxygen Consumption Rate (OCR) and Respiratory Chain Complexes Activities

The OCR in HCC cells was determined using XF96 Extracellular Flux Analyser (Seahorse Bioscience). The MitoTox Complete OXPHOS Activity Assay Kit (Abcam, #ab110419) was employed to measure the activities of the respiratory chain complexes. The OCR values were normalised to the cellular protein content.

### 2.13 Glucose Metabolism Intermediates Detection

Glucose metabolism intermediates were quantified utilising gas chromatography‐time of flight‐mass spectrometry (GC‐TOF‐MS). The measurements of these metabolic intermediates were normalised to cell number with the total amount of protein concentration.

### 2.14 Co‐Immunoprecipitation (Co‐IP) Assay

The protein samples from HCC cells (50 μL lysates) were combined with protein A beads that were conjugated with antibodies against LYRM2 (1:100) or HIF‐1α (1:50). These mixtures of cellular proteins and beads were rotated at 4°C overnight. Following centrifugation, the beads at the bottom of the tubes were washed with pre‐chilled lysis buffer (1 mL). Subsequent to the immunoprecipitation process, the protein samples were subjected to western blot to assess the protein interaction between HIF‐1α and LYRM2.

### 2.15 Statistical Analysis

The SPSS software (SPSS, Chicago, IL) was used for statistical analysis. Quantitative data from independent experiments are presented as the means ± standard error of the mean (SEM). Group differences were assessed using Student's t‐test for comparisons between two groups and one‐way ANOVA for comparisons among multiple groups. The Kaplan–Meier method was utilised to generate the survival curves stratified by LYRM2 levels. The survival curves and survival rates between groups categorised by LYRM2 levels were compared using the log‐rank test. A *p* value < 0.05 was considered to be statistically significant.

## 3 Results

### 3.1 LYRM2 Is Upregulated in HCC and Associated With patients' Unfavourable Clinical Features and Survival

**FIGURE 1:**
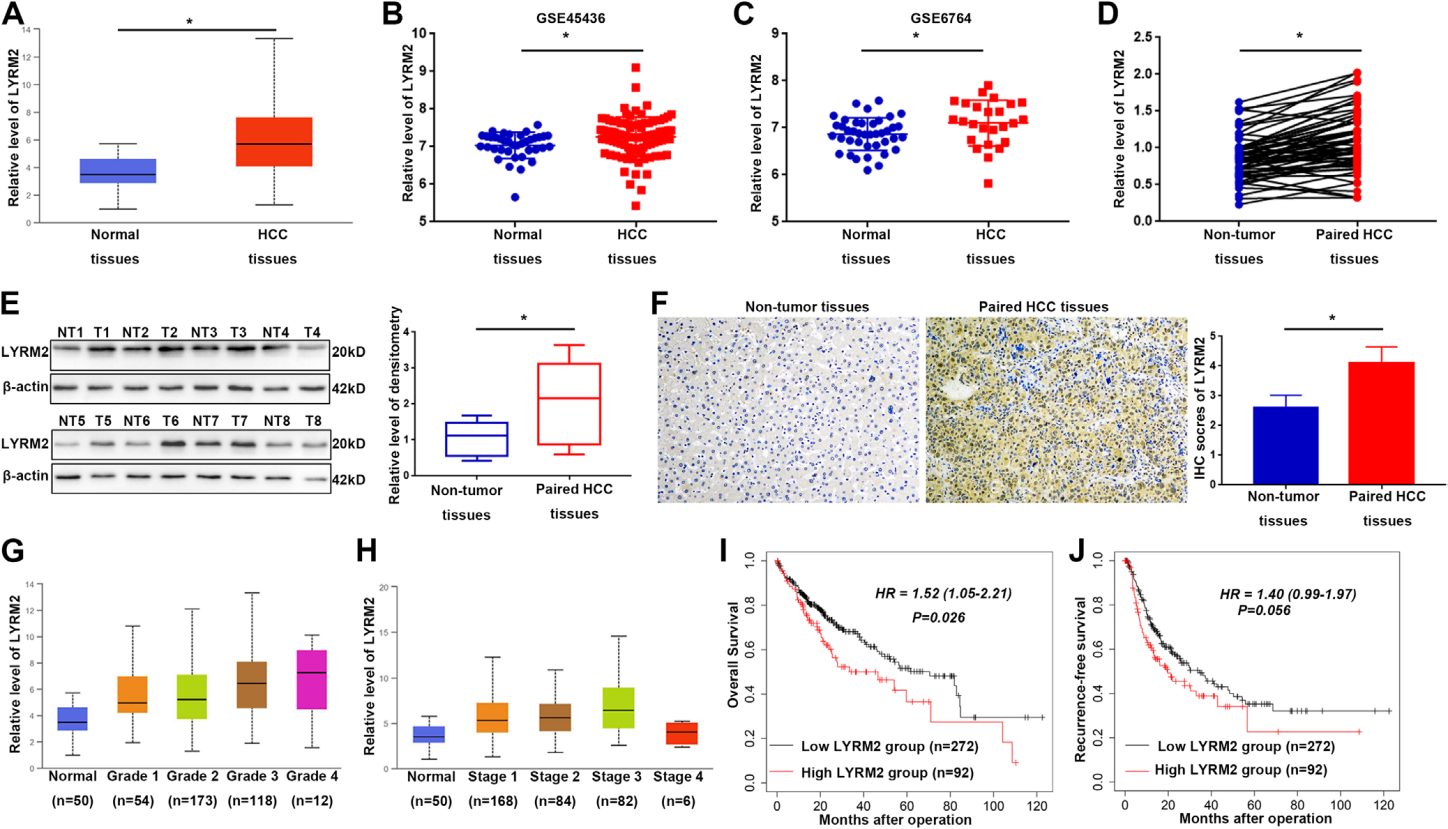
Upregulation of LYRM2 is frequently detected in HCC tissues and correlated with poor clinical features and prognosis of patients. (A) Relative LYRM2 expression level in HCC and non‐tumour liver tissues in TCGA database. (B, C) Relative LYRM2 expression level in two HCC datasets, GSE45436 and GSE6764. (D) mRNA level of LYRM2 was measured by qRT‐PCR in 63‐paired HCC tissues and adjacent non‐tumour liver tissues. (E) Protein level of LYRM2 was measured by western blot in 8‐paired HCC tissues and non‐tumour liver tissues. (F) IHC staining of LYRM2 staining in 63‐paired HCC and nontumor tissues. (G, H) Relative LYRM2 expression level in HCC tissues stratified by tumour grades and tumour stages. (I, J) prognostic value of LYRM2 expression was analysed in TCGA dataset of HCC. OS, overall survival; RFS, recurrence‐free survival. *, *p* < 0.05.

To explore the role of LYRM2 in HCC, we initially analysed the expression levels of LYRM2 in HCC tissues using TCGA databases. LYRM2 expression level was elevated in HCC tissues compared to adjacent non‐tumour liver tissues (Figure 1A). This upregulation of LYRM2 level in HCC was further corroborated by data obtained from GEO databases, specifically GSE45436 and GSE6764 (Figure 1B,C). To validate these findings using publicly available online databases, we measured LYRM2 expression levels in a cohort of paired HCC tissues and adjacent liver tissues. qRT‐PCR, western blot and IHC staining assays confirmed that LYRM2 was more abundant in HCC tissues (Figure 1D–F and Figure S1A, *p* < 0.05). Additionally, we observed that LYRM2 levels were markedly increased in HCC cell lines compared to MIHA cells (Figure S1B,C, *p* < 0.05). Furthermore, elevated LYRM2 levels were correlated with more advanced tumour grade (Figure 1G) and tumour stage (Figure 1H) in HCC patients, based on the data mining in TCGA database. Correlation analysis between LYRM2 expression status and clinical data from our patient cohort (Table 1) indicated that positive staining for LYRM2 in HCC tissues was correlated with liver cirrhosis (*p* = 0.041), larger tumour diameter (*p* = 0.024), the presence of multiple tumour nodules (*p* = 0.012) and microvascular invasion (*p* = 0.019). Notably, high LYRM2 levels in HCC patients were associated with a lower overall survival rate (Figure 1I, *p* = 0.026) and a reduced recurrence‐free survival rate (Figure 1J, *p* = 0.056).

**TABLE 1:** Clinicopathological features of HCC patients according to LYRM2 expression.

Variables	LYRM2 positive	LYRM2 negative	*p*
	(*n* = 28)	(*n* = 35)	
Age			
≤ 60	11	16	0.798
> 60	17	19	
Gender			
Female	4	8	0.523
Male	24	27	
Cirrhosis			
Negative	7	18	0.041
Positive	21	17	
HBV infection			
Negative	9	10	0.789
Positive	19	25	
AFP			
≤ 200 ng/mL	8	15	0.298
> 200 ng/mL	20	20	
Tumour size			
≤ 5 cm	10	23	0.024
> 5 cm	18	12	
Tumour number			
Single	10	24	0.012
Multiple	18	11	
Microscopic vascular invasion			
Negative	12	26	0.019
Positive	16	9	
Edmondson–Steiner grade			
1–2	19	29	0.235
3–4	9	6	

### 3.2 LYRM2 Promotes Proliferation, Metastasis, EMT and AKT Phosphorylation of HCC Cells In Vitro

**FIGURE 2:**
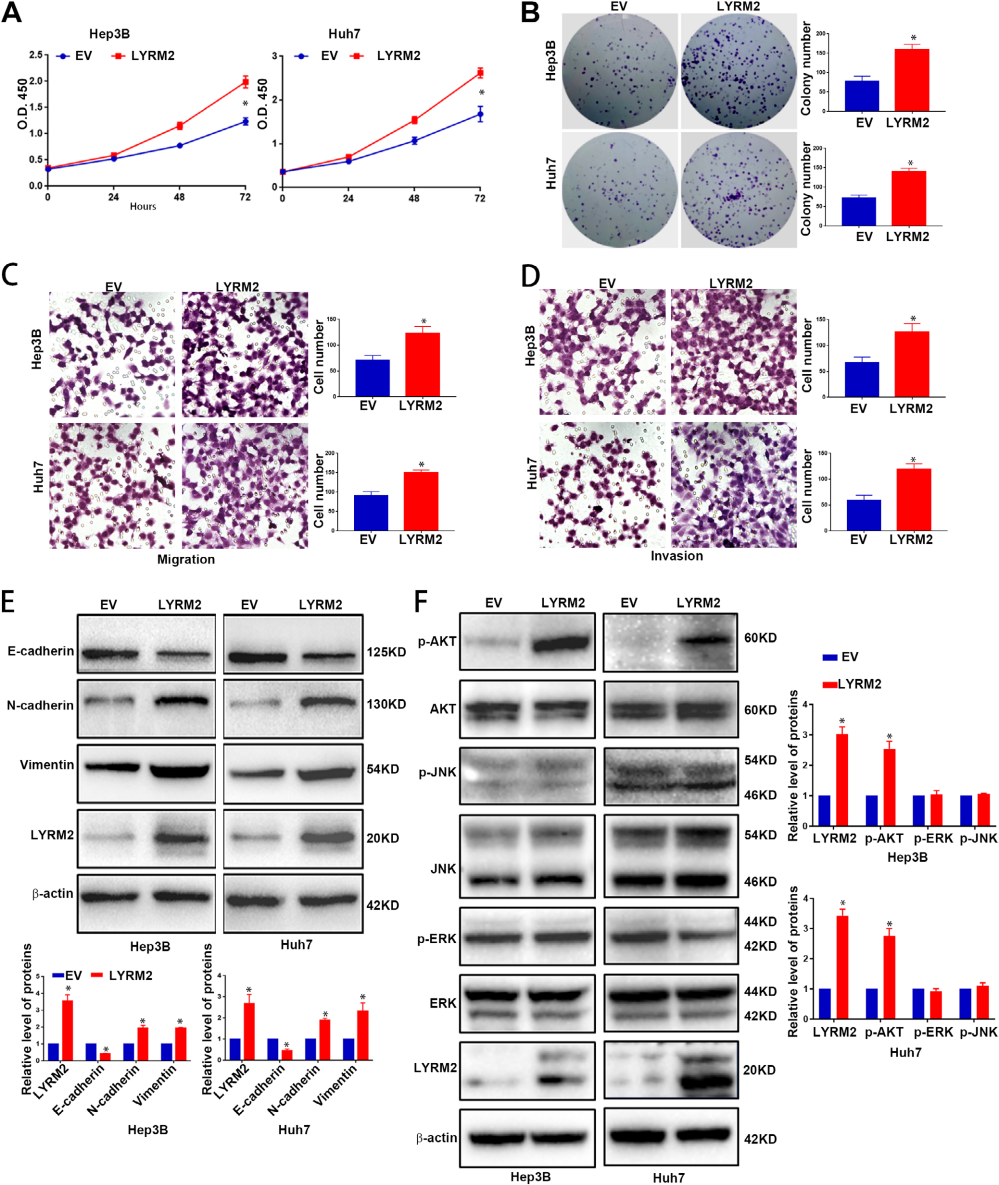
Overexpression of LYRM2 promoted the proliferation, metastasis, EMT and AKT pathway activation of HCC cells. (A, B) The influence of LYRM2 overexpression on cell viability (CCK8 assay) and colony formation ability was determined in Hep3B and Huh7 cells. (C, D). The influence of LYRM2 overexpression on cell migration and invasion were determined in Hep3B and Huh7 cells by Transwell assays. (E, F) Effects of LYRM2 overexpression on the expression level of EMT makers (E‐cadherin, N‐cadherin and Vimentin) and classical pathways activation (JNK, ERK and AKT pathways) were evaluated in Hep3B and Huh7 cell by western blot. *, *p* < 0.05.

**FIGURE 3:**
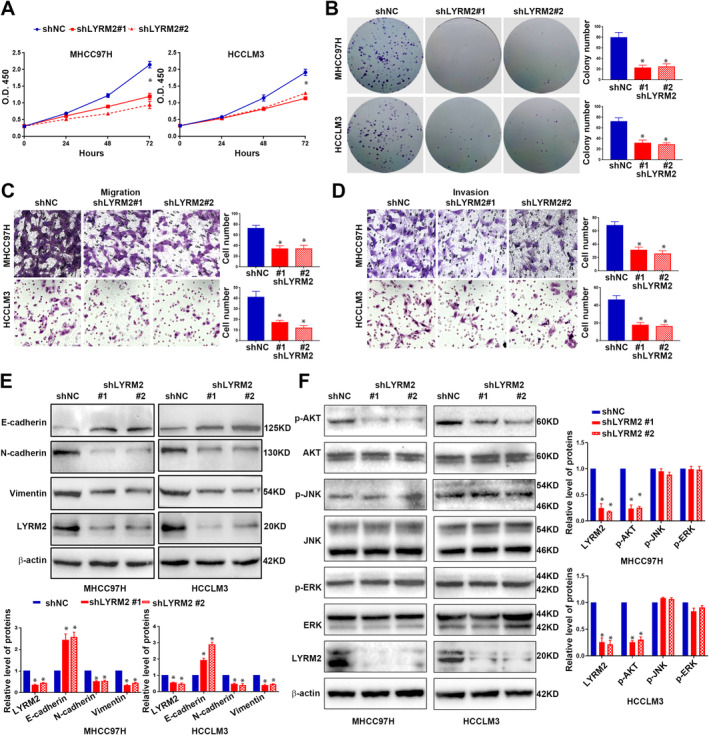
Knockdown of LYRM2 suppressed the proliferation, metastasis, EMT and AKT pathway activation of HCC cells. (A, B) The influence of LYRM2 knockdown on cell viability (CCK8 assay) and colony formation ability was determined in MHCC97H and HCCLM3 cells. (C, D) The influence of LYRM2 knockdown on cell migration and invasion were determined in MHCC97H and HCCLM3 cells by Transwell assays. (E, F) The influence of LYRM2 knockdown on the level of EMT makers (E‐cadherin, N‐cadherin and Vimentin) and classical pathways activation (JNK, ERK and AKT pathways) were evaluated in MHCC97H and HCCLM3 cells by western blot. *, *p* < 0.05.

To investigate the role of LYRM2 in HCC cells, we established cell lines with stable overexpression of LYRM2 in Hep3B (Figure S2A,B) and Huh7 (Figure S2C,D), as well as cell lines with LYRM2‐knockdown inMHCC97H (Figure S3A,B) and HCCLM3 (Figure S3C,D). The forced expression of LYRM2 significantly promoted proliferation (Figure 2A) and colony formation of Hep3B and Huh7 cells (Figure 2B). To assess the impact of LYRM2 on metastatic ability, Transwell assay showed that LYRM2 overexpression resulted in an increased number of Hep3B and Huh7 cells that migrated or invaded through the membrane or Matrigel (Figure 2C,D). Given EMT is associated with the enhanced metastatic capacities of cancer cells [17], we further explored whether LYRM2 affects the EMT process in HCC cells. Overexpression of LYRM2 decreased the level of E‐cadherin, while increased the levels of N‐cadherin and vimentin (Figure 2E and Figure S4A). The activation or inhibition of canonical signalling pathways, including AKT, ERK and JNK, is responsible for the enhanced growth and metastasis of tumour cells [18, 19]. In this regard, we explored the activation status of the AKT, ERK and JNK pathways following LYRM2 overexpression. Western blot revealed a significant increase in AKT phosphorylation of AKT after ectopic expression of LYRM2, whereas the phosphorylation levels of ERK and JNK remained unchanged (Figure 2F). Conversely, the knockdown of LYRM2 in HCCLM3 and MHCC97H cells resulted in decreased the cell viability (Figure 3A), proliferation (Figure 3B), migration (Figure 3C) and invasion (Figure 3D). Following LYRM2 knockdown, the expression of E‐cadherin was increased, while the levels of N‐cadherin and vimentin level decreased (Figure 3E and Figure S4B). Additionally, LYRM2 knockdown reduced the phosphorylation of AKT in HCC cells (Figure 3F).

### 3.3 LYRM2 Knockdown Inhibits the Growth and Metastasis of HCC Cells In Vivo

**FIGURE 4:**
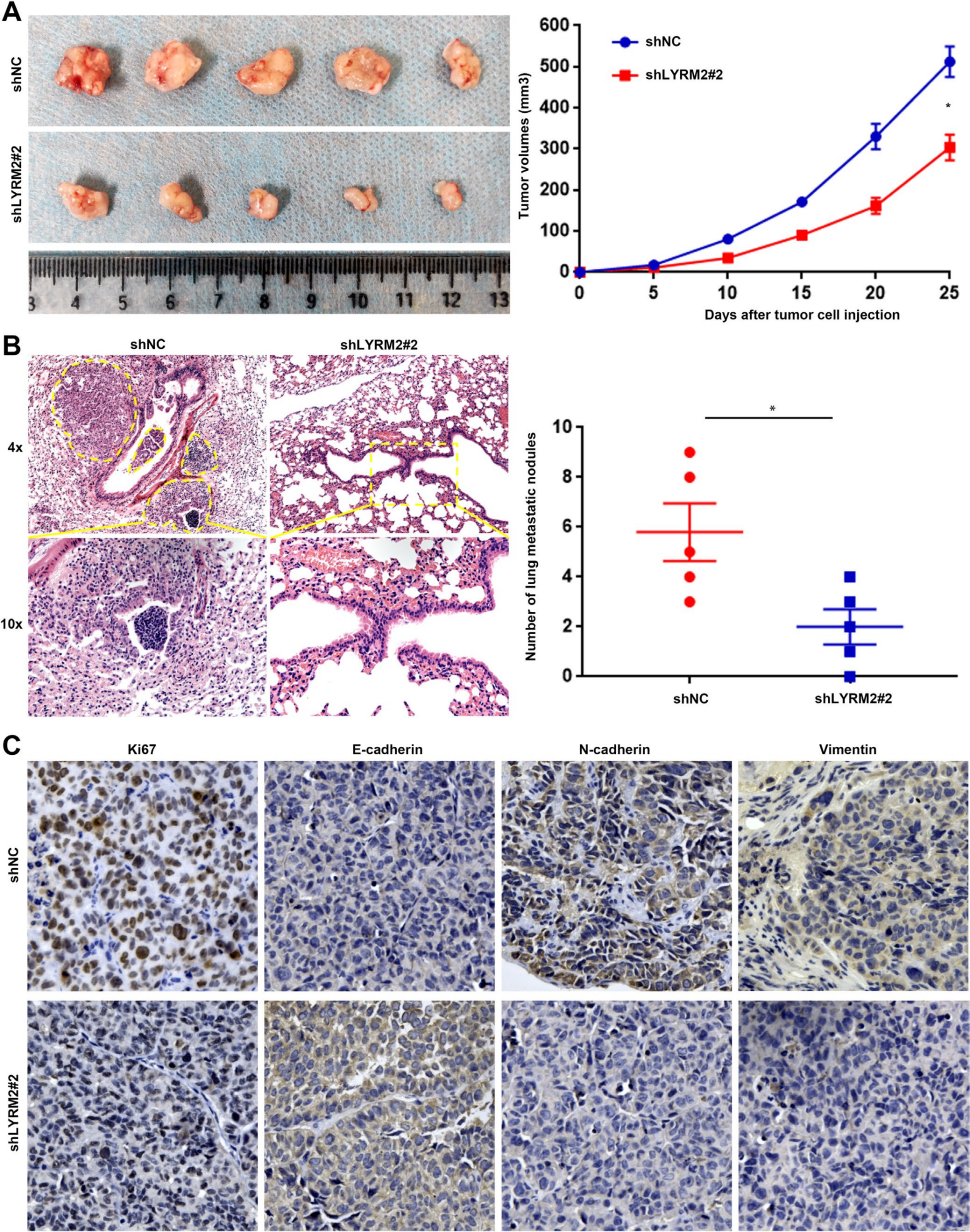
Knockdown of LYRM2 suppressed the growth and lung metastasis of HCC cells in nude mice. (A) Subcutaneous implantation model was established to evaluate the effect of LYRM2 knockdown on the growth ability of HCCLM3 in nude mice. Tumour size was compared between NC shRNA and LYRM2 shRNA groups. (B) Tail vein injection model was established to determine the effect of LYRM2 knockdown on the metastatic ability of HCCLM3 in nude mice. The number of lung metastatic nodules was compared between NC shRNA and LYRM2 shRNA groups. (C) IHC staining for Ki67 and EMT makers (E‐cadherin, N‐cadherin and Vimentin) was used to demonstrate the effect of LYRM2 knockdown on the proliferation and EMT of HCCLM3 cell in xenograft tumours. *, *p* < 0.05.

To verify the functional role of LYRM2 in vivo, we constructed both a subcutaneous xenograft model and a tail vein injection model. In the subcutaneous xenograft models, the volume of subcutaneous tumours formed in the LYRM2 knockdown group was significantly smaller compared to the control group (Figure 4A). In the tail vein injection model, mice in the LYRM2 knockdown group exhibited a reduced number of lung metastatic nodules relative to those in the control group (Figure 4B). Ki67 staining revealed a decreased percentage of Ki67‐positive cells in the LYRM2 knockdown group (Figure 4C). Additionally, IHC staining demonstrated LYRM2 knockdown resulted in an increased level of E‐cadherin, while decreasing the expression of N‐cadherin and vimentin in the tumour tissues (Figure 4C). These indicate LYRM2 knockdown inhibits the growth, metastasis and EMT of HCC cells in vivo.

### 3.4 LYRM2 Promotes Aerobic Glycolysis and Inhibits Mitochondrial Respiration in HCC Cells

**FIGURE 5:**
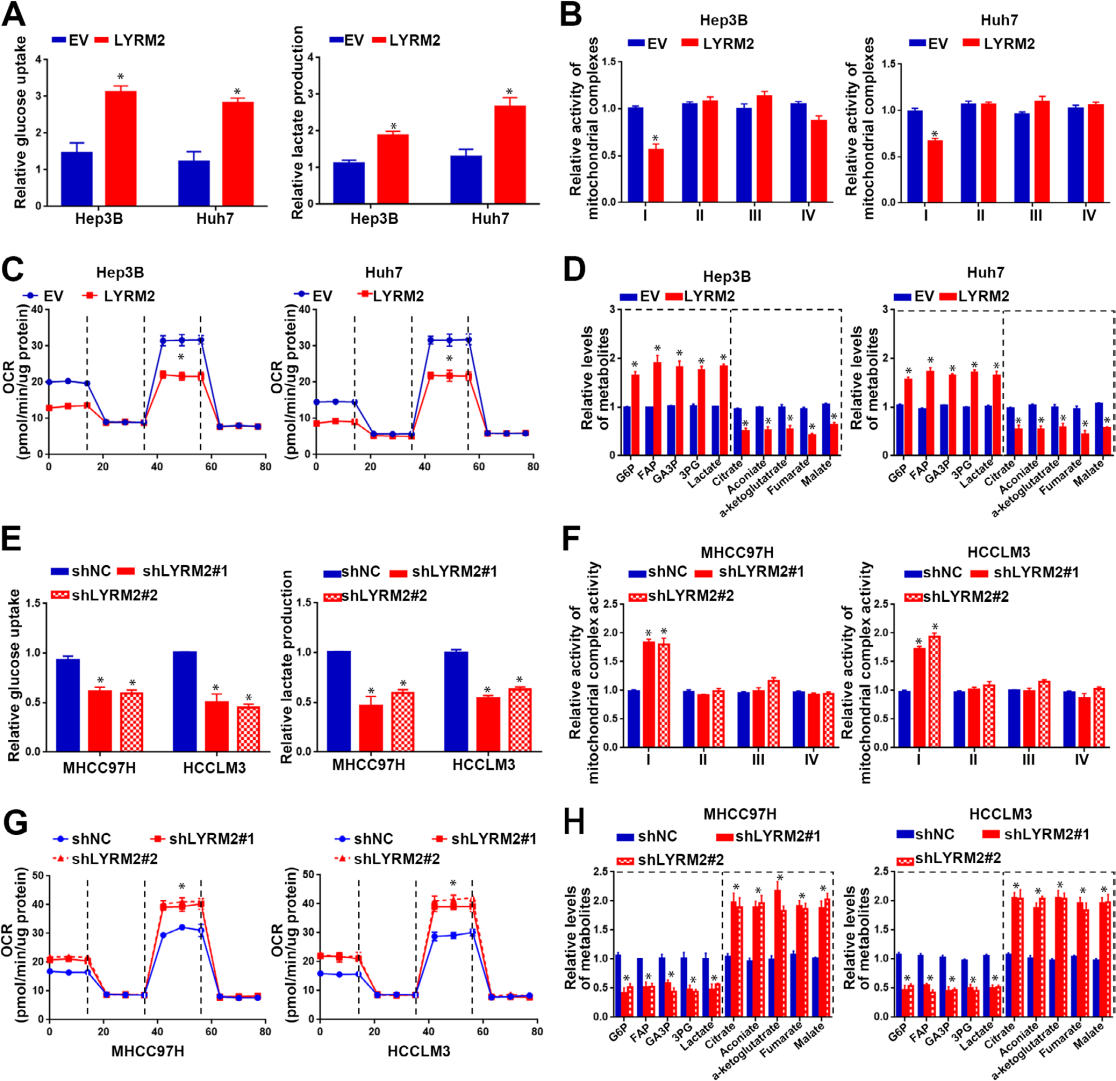
LYRM2 promotes aerobic glycolysis in HCC cells. (A) The influence of LYRM2 overexpression on glucose uptake and lactate production was determined in Hep3B and Huh7 cells. (B, C) Effects of LYRM2 overexpression on OXPHOS complexes activities (B) and mitochondrial oxygen consumption rate (C) were determined in Hep3B and Huh7 cells. (D) Metabolites involved in aerobic glycolysis and TCA cycle were measured in Hep3B and Huh7 cells after LYRM2 overexpression. (E) The influence of LYRM2 knockdown on the glucose uptake and the lactate production was determined in MHCC97H and HCCLM3 cells. (F, G) Effects of LYRM2 knockdown on OXPHOS complexes activities (F) and mitochondrial oxygen consumption rate (G) were determined in MHCC97H and HCCLM3 cells. (H) Metabolites involved in glycolysis and TCA cycle were measured in MHCC97H and HCCLM3 cells after LYRM2 knockdown. *, *p* < 0.05.

**FIGURE 6:**
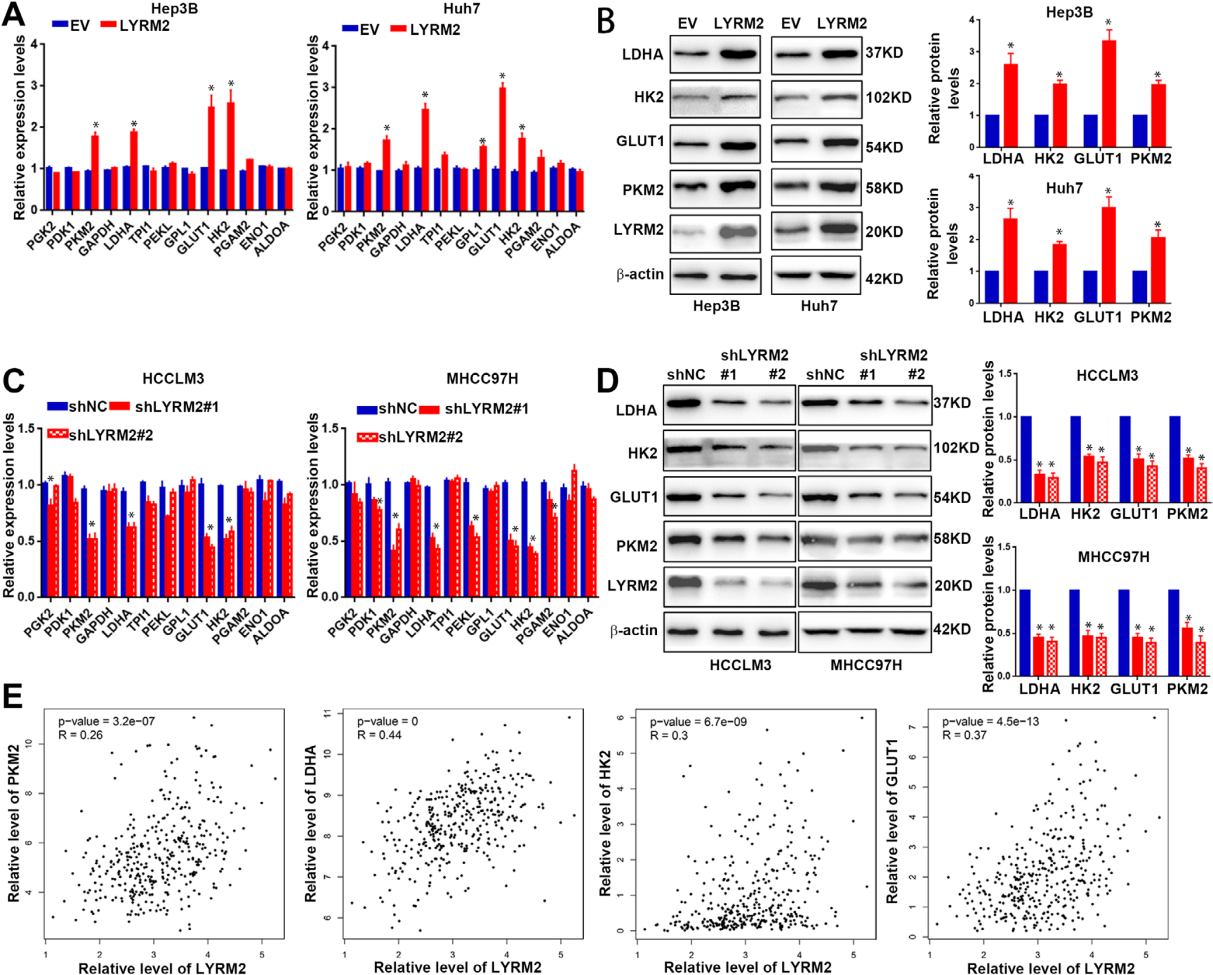
LYRM2 facilitated glycolysis via promoting the glycolytic gene expressions. (A) The effect of LYRM2 overexpression on the mRNA levels of glycolytic genes (ALDOA, ENO1, HK2, GAPDH, GLUT1, GPL‐1, LDHA, PDK1, PEKL, PGAM2, PGK2, PKM2, TPI1) was measured by qRT‐PCR analysis. (B) The influence of LYRM2 overexpression on the expression levels of LDHA, HK2, GLUT1 and PKM2 proteins was measured by western blot. (C) The effect of LYRM2 knockdown on the mRNA levels of glycolytic genes (ALDOA, ENO1, HK2, GAPDH, GLUT1, GPL‐1, LDHA, PDK1, PEKL, PGAM2, PGK2, PKM2 and TPI1) was measured by qRT‐PCR analysis. (D) The effect of LYRM2 knockdown on the protein levels of LDHA, HK2, GLUT1 and PKM2 was measured by western blot. (E) Correlation analysis for the expression level of LYRM2 and the expression levels of LDHA, HK2, GLUT1 and PKM2 based on the data in TCGA database. *, *p* < 0.05.

Next, we investigated the role of LYRM2 in the metabolic reprogramming of HCC cells, given the increased glycolysis in cancer cells is a well‐recognised hallmark responsible for malignant growth and metastasis [3]. LYRM2 overexpression increased glucose uptake and lactate production (Figure 5A), suggesting a reduction in aerobic glycolysis in Hep3B and Huh7 cells. In light of the negative feedback mechanism between cellular glycolysis and mitochondrial respiration [5], we further investigated the effects of LYRM2 on mitochondrial oxidative phosphorylation. Analysis of the activities of OXPHOS complexes showed that LYRM2 overexpression inhibited the activity of complex I (Figure 5B), while the activities of Complex II–IV remained unchanged (Figure 5B). Additionally, a decrease in the OCR was observed in Hep3B and Huh7 cells following LYRM2 overexpression (Figure 5C). We also conduct a metabolomics analysis subsequent to the forced expression of LYRM2 in HCC cells, which demonstrated an increase in glycolytic metabolites and a decrease in metabolites associated with TCA cycle upon LYRM2 overexpression (Figure 5D). Conversly, knockdown of LYRM2 resulted in opposite effects on glucose uptake, lactate production, complex I activity, OCR, and level of glycolytic and metabolites (Figure 5E–H). Consistent with these functional outcomes, LYRM2 everexpression was found to elevate the mRNA and protein levels of key metabolic enzymes involved in glucose metabolism, including PKM2, LDHA, HK2 and GLUT1 (Figure 6A,B). In contrast, LYRM2 knockdown led to reduction in the the levels of these metabolic enzymes (Figure 6C,D). Furthermore, correlation analysis utilising on TCGA databases indicated positive associations between LYRM2 levels and these key regulators of glycolysis, including PKM2, LDHA, HK2 and GLUT1 (Figure 6E). Taken together, LYRM2 participates in the metabolic reprogramming of HCC cells by promoting aerobic glycolysis and inhibiting mitochondrial OXPHOS.

### 3.5 LYRM2 Regulates Glucose Metabolism Reprogramming by Enhancing the Stability of HIF‐1α Protein

**FIGURE 7:**
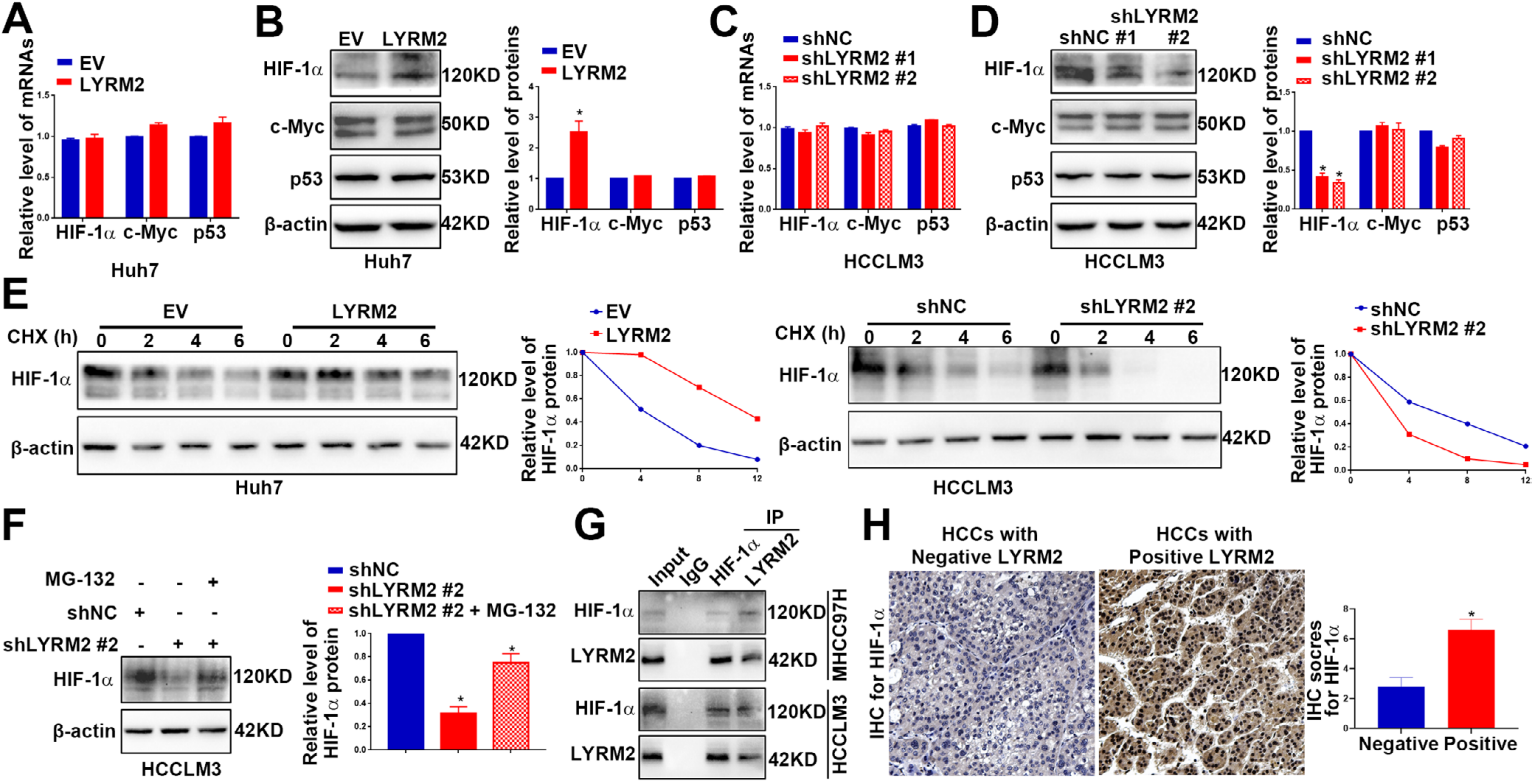
LYRM2 upregulated HIF‐1a protein level by enhancing its protein stability. (A, B) The effects of LYRM2 overexpression on the mRNA and protein level of c‐MYC, HIF‐1a and P53 were measured in Huh7 cells by qRT‐PCR and western blot analysis. (C, D) The effects of LYRM2 knockdown on the mRNA and protein level of c‐MYC, HIF‐1a and P53 were measured in HCCLM3 cells by qRT‐PCR and western blot analysis. (E) The effect of LYRM2 overexpression or knockdown on the protein stability of HIF‐1a was evaluated by western blot. HCC cells were treated with cycloheximide (CHX, 10 μg/mL) for indicated time length. (F) HCCLM3 cells were treated with MG132 (10 μM) for 2 h to block proteasomal degradation. Western blot was performed to determine whether MG132 (200 μM) prevented the HIF‐1a protein from degradation induced by LYRM2 knockdown. (G) Co‐IP was performed to determine the interaction between LYRM2 protein and HIF‐1a protein in the cellular extracts from HCCLM3. (H) The level of HIF‐1a protein in HCC specimens was compared between HCC tissues with negative and positive staining LYRM2. *, *p* < 0.05.

Classical transcriptional regulators, including HIF‐1α, p53 and c‐Myc, play a crucial role in modulating both glycolysis and OXPHOS [7, 8, 9, 10]. To clarify the molecular mechanisms by which LYRM2 modulate the glucose reprogramming, we assessed the effects of LYRM2 on the expression levels of these critical regulators. Upon overexpression of LYRM2, the protein level of HIF‐1α was increased (Figure 7A), while its mRNA level remained unchanged (Figure 7B). In contrast, the expression of p53 and c‐Myc expression was not significantly altered eithor the mRNA or protein levels (Figure 7A,B). Conversely, LYRM2 knockdown resulted in a decrease in the protein level of HIF‐1α, with no significant effect on HIF‐1α mRNA levels (Figure 7C,D). Furthermore, an analysis of the TCGA database revealed a positive correlation between the levels of HIF‐1α and LYRM2 (Figure S5). However, neither LYRM2 overexpression nor LYRM2 knockdown had any effect on HIF‐2α protein level (Figure S6). IHC staining of subcutaneous tumour tissues indicated that the protein level of HIF‐1α was reduced in the LYRM2 konckdown group (Figure S7). The levels of P53, c‐Myc and HIF‐2α proteins were not significantly decreased in the tumour tissues derived from HCCLM3 cells with LYRM2 knockdown (Figure S7).

**FIGURE 8:**
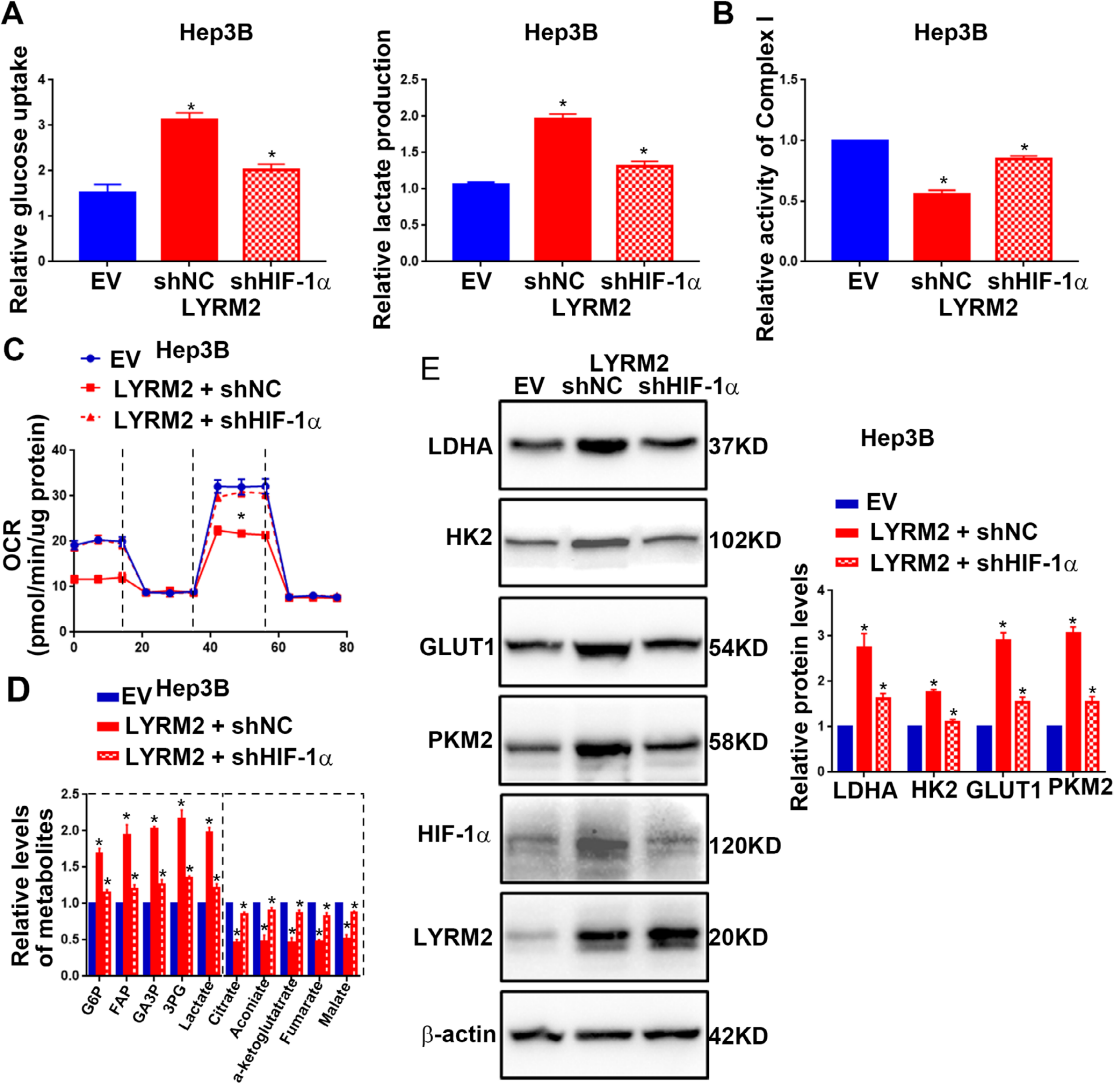
HIF‐1α knockdown blocked the promoting effect of LYRM2 overexpression on aerobic glycolysis. Hep3B cells in control group or LYRM2 overexpression group were transfected with NC shRNA or HIF‐1α shRNA. (A–E) The changes of (A) gucose uptake and lactate production, (B) activity of mitochondrial complex I, (C) mitochondrial oxygen consumption rate, (D) metabolites involved in glycolysis and TCA cycle and (E) protein levels of LDHA, HK2, GLUT1 and PKM2 were measured in Hep3B cells with different treatment as indicated. *, *p* < 0.05.

The expression of HIF‐1α expression was found to be altered solely at the protein level leading us to hypothesise that LYRM2 may increase HIF‐1α protein level by enhancing its stability. As illustrated in Figure 7E, the overexpression of LYRM2 significantly delayed the degradation process of HIF‐1α. Conversely, the knockdown of LYRM2 in HCC cells increased HIF‐1α degradation (Figure 7E). Treatment of HCCLM3 cells with the proteasome inhibitor MG‐132 effectively rescued HIF‐1α protein from degradation induced by LYRM2 knockdown (Figure 7F). These findings suggest that LYRM2 enhances the protein level of HIF‐1α by preventing its degradation via the proteasome pathway. Co‐IP assays showed that LYRM2 interacted with HIF‐1α (Figure 7G). IHC staining demonstrated that HCC tissues positive with LYRM2 protein exhibited elevated levels ofHIF‐1α protein (Figure 7H). Functionally, the transfection of HIF‐1α shRNA effectively abolished HIF‐1α expression in HCC cells, and subsequently inhibiting the glucose metabolism reprogramming induced by LYRM2 overexpression (Figure 8A–E).

### 3.6 Enhanced Glycolysis Facilitates the Tumour‐Promoting Effects of LYRM2 in HCC

**FIGURE 9:**
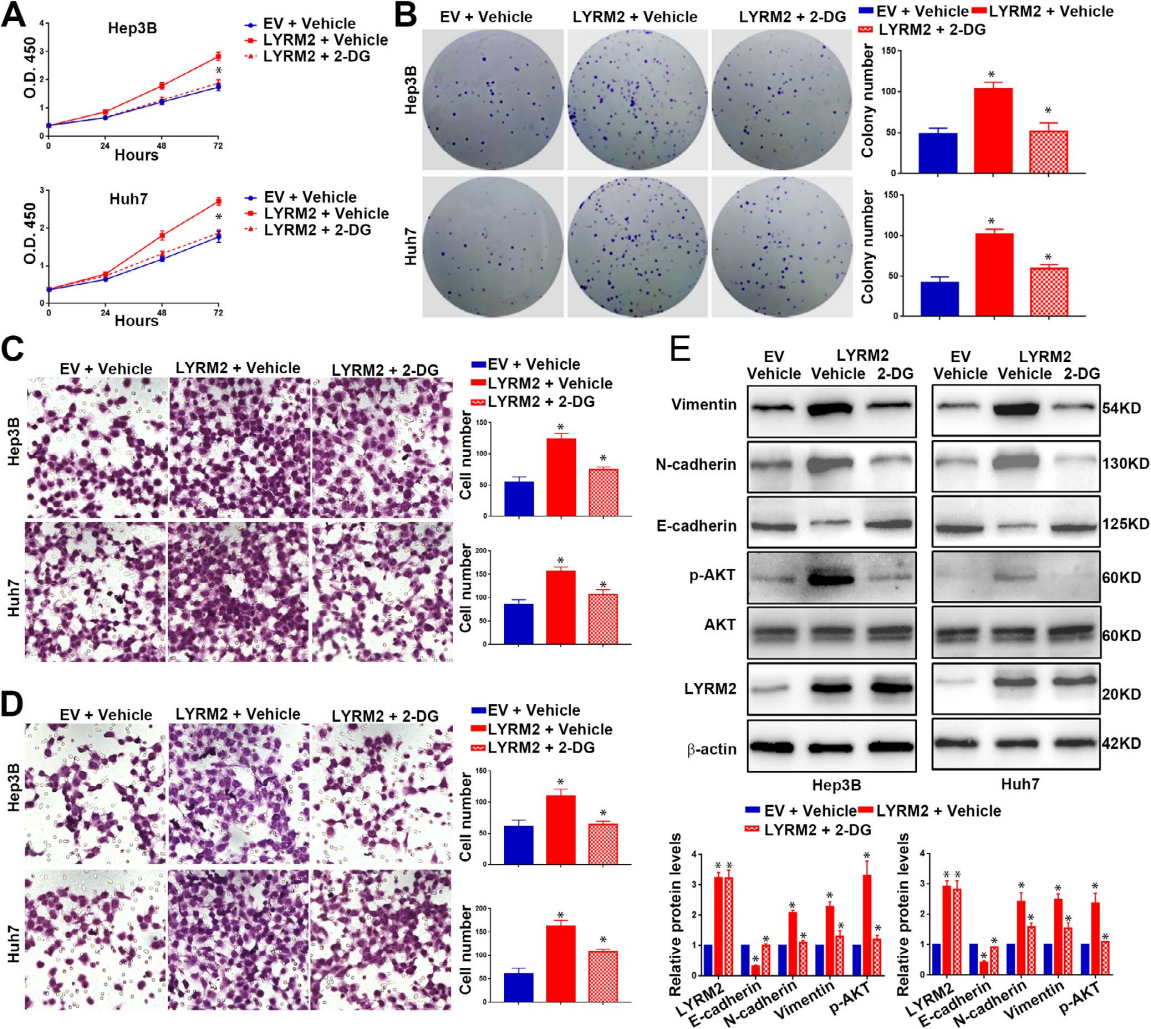
LYRM2 promotes the proliferation, migration, invasion and EMT of HCC cells through mediating metabolic reprogramming. Hep3B or Huh7 cells in control group or LYRM2 overexpression group were treated with 2‐DG. A‐E The changes of (A) cell viability (CCK8 assay), (B) colony formation, (C) cell migration, (D) cell invasion and (E) the expression level of EMT makers (E‐cadherin, N‐cadherin and Vimentin) and AKT phosphorylation were determined in HCC cells with indicated treatments. *, *p* < 0.05.

To determine the glucose metabolic reprogramming induced by LYRM2, which is critical for the increased tumour growth and metastasis, Hep3B and Huh7 cells with LYRM2 overexpressing were treated with 2‐deoxy‐d‐glucose (2‐DG), a known inhibitor of cellular glycolysis. The administration of 2‐DG effectively inhibited the enhanced proliferation (Figure 9A), colony formation (Figure 9B), migration (Figure 9C) and invasion (Figure 9D) associated with LYRM2 overexpression. Additionally, 2‐DG treatment in Hep3B and Huh7 cells suppressed EMT and the phosphorylation of AKT induced by LYRM2 overexpression (Figure 9E). In all, these findings suggest that the metabolic shift towards aerobic glycolysis is crucial for the proliferation, invasion, migration, EMT and AKT activation driven by LYRM2.

## 4 Discussion

LYRM2 is a member of LYRMs family that is evolutionarily conserved and plays a regulatory role in mitochondrial metabolism [20, 21, 22]. Abnormal expression of LYRMs has been linked to the onset and progression of breast cancer and oesophageal squamous cell carcinoma [13, 14, 15]. Previous study demonstrated that LYRM2 expression is increased in colorectal cancer, where it promotes the growth of cancer cells [16]. In this study, we demonstrate that LYRM2 expression is increased in HCC tissues and cell lines. This upregulation of LYRM2 is correlated with poor prognosis and unfavourable clinical characteristics in patients with HCC, suggesting that LYRM2 contributes to the progression of HCC. To investigate the functional impact of LYRM2 on HCC cells, we conducted experiments involving both overexpression and knockdown of LYRM2. The forced expression of LYRM2 in HCC ells markedly enhanced cell proliferation, colony formation, migration and metastasis. Conversely, the knockdown of LYRM2 markedly decreased cell proliferation and metastatic abilities. Given that EMT is an important reason for the enhanced cell metastasis [23], we checked the role of LYRM2 in the EMT process of cancer cells. Our finding revealed that overexpression of LYRM2 decreased the level of epithelial marker while increased the level of mesenchymal markers, indicating that LYRM2 promotes HCC metastasis through regulating EMT phenotype. These data were further corroborated by the subcutaneous implantation and tail vein injection models, which revealed that HCC cells with LYRM2 knockdown exhibited reduced the subcutaneous tumour volumes and a lower rate and number of lung metastasis compared to those from the control group. Additionally, Ki‐67 and IHC staining for E‐cadherin, N‐cadherin and Vimentin in xenograft tumours revealed that the suppression of LYRM2 in HCC cells inhibited both the cell proliferation and EMT. The AKT pathway, JNK pathway and ERK pathway are classical pathways that regulate cancer growth, metastasis and EMT [18, 19]. In this study, we found that LYRM2 enhances AKT phosphorylation in HCC cells, while activation status of ERK and JNK remained unchanged following LYRM2 knockdown or overexpression. This suggests that the activation of the AKT is responsible for the promoting effects of LYRM2 on cancer growth and metastasis. However, it is noteworthy that the overexpression of LYRM2 did no significantly enhance tumour growth and metastasis in vivo (data not shown). The following reasons may account for for this observation. First, as an oncogene that is overexpressed in HCC, the impact of LYRM2 knockdown in HCC cells on the in vivo growth and metastasis may be more pronounced than that of LYRM2 overexpression. Second, the tumour microenvironment (e.g., hypoxia) may induce the expression of LYRM2 in HCC cells in control group during the process of in vivo growth and metastasis, potentially diminishing the effects of LYRM2 overexpression. This area warrants further investigation in the future.

Cancer cells exhibit a preference for reprogramming glucose metabolism from mitochondrial respiration to aerobic glycolysis, even in the presence of sufficient oxygen. This shift generates abundant glycolytic metabolites and adenosine triphosphate (ATP), which faciliates enhanced cellular proliferation and motility [24]. The reprogramming of glucose metabolism is mediated is achieved by the upregulation of glycolytic enzymes, including HK2, PKM2 and LDHA [25]. We observed that LYRM2 enhanced glucose uptake and lactate production while simultaneously decreasing mitochondrial respiration. The expression of key glycolytic enzymes is elevated by LYRM2, whereas the complex I activity is inhibited. Notably, in the context of colorectal cancer, LYRM2 has been shown to promote OXPHOS of colorectal cancer cells [16]. These indicate the influence of LYRM2 on cancer cell metabolism is dependent on the specific cancer type. Previous studies have demonstrated that the reprogramming of glucose metabolism from mitochondrial respiration to aerobic glycolysis is essential for the rapid growth, metastasis and EMT phenotype observed in cancer cells [26, 27, 28, 29]. In the present study, the inhibition of glycolysis using 2‐DG blocked the enhanced proliferation, metastasis and AKT phosphorylation induced by LYRM2 overexpression. These results indicate that LYRM2‐induced glucose reprogramming and glycolysis are critical for the oncogenic effects of LYRM2 in HCC cells.

HIF‐1α, p53 and c‐Myc are well‐established as critical regulators of glucose metabolism, influencing genes involved in cellular glycolysis and mitochondrial oxidative phosphorylation [30, 31]. The present study demonstrates that the overexpression of LYRM2 resulted in an increased level of HIF‐1α protein, while it dose no affect the levels of P53 and c‐Myc. As a key regulator of the Warburg effect, HIF‐1α facilitates the transcription of glycolytic regulatory genes, shifts cellular glucose metabolism from oxidative phosphorylation to aerobic glycolysis and inhibits mitochondrial respiration [10]. In our investigation, the knockdown of HIF‐1α blocked the promoting effect of LYRM2 on the Warburg effect, as evidenced by decreased glycolysis and enhanced mitochondrial respiration. These findings suggest that LYRM2 modulates metabolic reprogramming by elevating the protein level of HIF‐1α. In patients with HCC, increased levels of HIF‐1α are correlated with poor prognosis [32] and contribute to sorafenib resistance [33]. Under normoxic conditions, the hydroxylation of proline residues and acetylation of lysine residue on HIF‐1α lead to its interaction with E3 ligase, resulting in ubiquitin–proteasomal degradation of HIF‐1α [34]. Our study reveals that LYRM2 increases the protein level of HIF‐1α without affecting its mRNA levels. Inhibition of proteasomal degradation using MG‐132 mitigated the decrease of HIF‐1α protein levels caused by LYRM2 knockdown. These data indicate that LYRM2 diminishes proteasomal degradation and enhances the stability of HIF‐1α protein. This regulatory influence of LYRM2 on HIF‐1α protein was further validated by the positive correlation observed between HIF‐1α and LYRM2 protein levels in clinical samples from HCC patients. However, several questions regarding the regulatory relationship between HIF‐1α and LYRM2, as well as the therapeutic potential of the LYMR2/HIF‐1α axis, remain unresolved and require further validation in the future: (1) whether overexpression of HIF‐1α can counteract the inhibitory effect of LYRM2 knockdown on cellular glycolysis; (2) whether this regulatory interaction between LYRM2 and HIF‐1α can be validated in vivo; (3) the specific molecular mechanism through which LYRM2 reduces the degradation of HIF‐1α; (4) whether targeting the LYRM2/HIF‐1α axis (using HIF‐1a flox mice or LYRM2 flox mice models) can impede the development of HCC. Previous study has indicated that HIF‐2α is also a critical protein that responds directly to hypoxia stress and activates the expression of various glycolysis‐related genes [35]. Our data further indicate that LYRM2 does not influence the level of HIF‐2α protein, suggesting that the regulatory effect of LYRM2 on metabolic reprogramming is specifically dependent on HIF‐1α rather than HIF‐2α.

**FIGURE 10:**
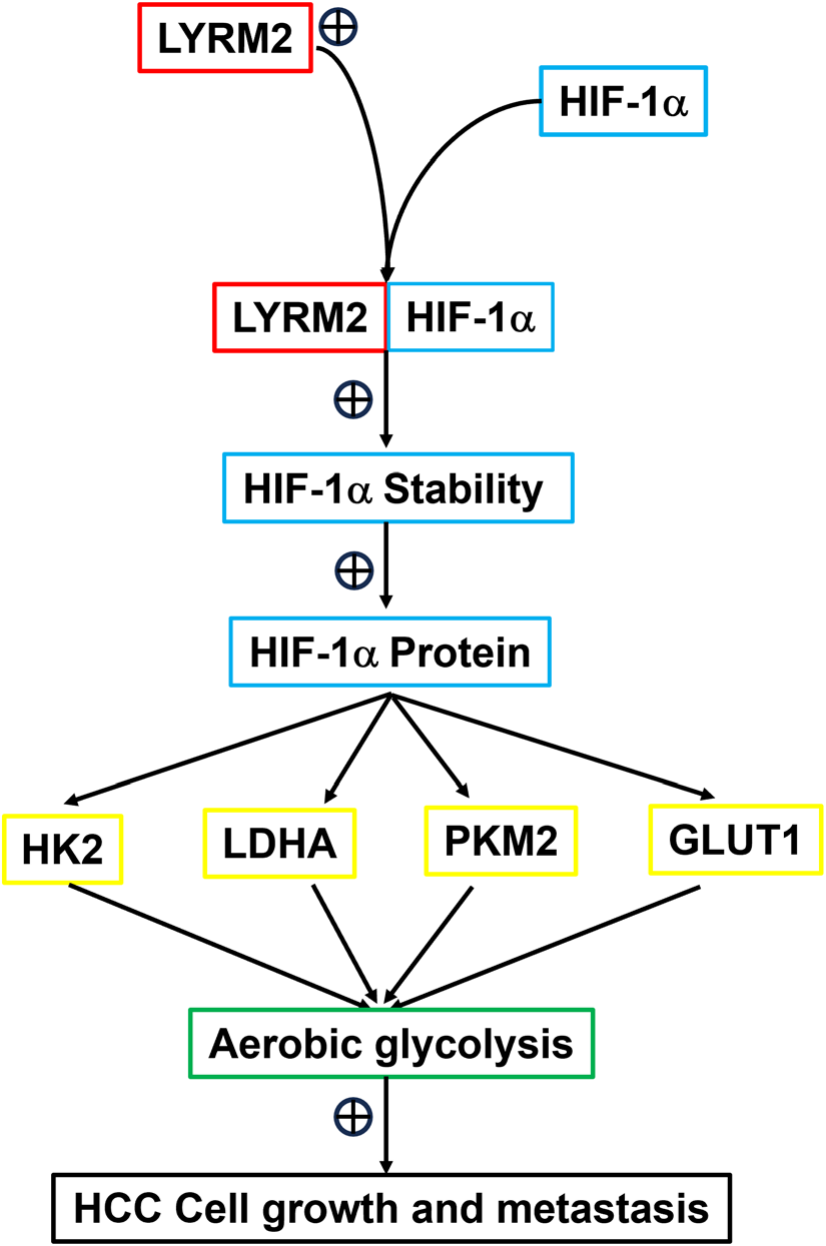
The schematic graph for the molecular mechanism of LYRM2 in promoting the growth and metastasis of HCC. LYRM2 interacts with HIF‐1α and increases the stability of HIF‐1α protein, leading to elevated HIF‐1α protein levels in HCC cells. Activated LYRM2/HIF‐1α axis increases the expression of key glycolytic enzymes (HK2, LDHA, PKM2 and GLUT1), activates cellular glycolysis and results in HCC growth and metastasis.

This study collectively demonstrates that LYRM2 is overexpressed in HCC. Functionally, LYRM2 promotes both the growth and metastasis of HCC. Mechanistically, LYRM2 exerts its tumour‐promoting effects in HCC by enhancing glycolysis while inhibiting the mitochondrial respiration. Furthermore, the findings indicate that LYRM2 promotes glucose reprogramming by enhancing the protein stability of HIF‐1α (Figure 10). This study suggests that LYRM2 can potentially serve as both a biomarker and a therapeutic target for HCC.

## Author Contributions

**Bingfu Fan:** data curation (equal), formal analysis (equal), methodology (equal), software (equal), validation (equal), visualization (equal), writing – original draft (lead). **Yueqin Zhang:** data curation (equal), formal analysis (equal), methodology (equal), software (equal), validation (equal), visualization (equal). **Lu Zhou:** formal analysis (equal), funding acquisition (equal), methodology (equal), software (equal), validation (equal), visualization (equal). **Zhongchun Xie:** methodology (equal), software (equal). **Jie Liu:** funding acquisition (equal), investigation (equal), methodology (equal), supervision (equal), validation (equal). **Chengwu Zhang:** funding acquisition (supporting), investigation (supporting), resources (supporting). **Changwei Dou:** conceptualization (lead), project administration (lead), writing – review and editing (lead).

## Ethics Statement

The present study was reviewed and approved by the Ethics Committee of Zhejiang Provincial People's Hospital (approval nos. 2023310 and 2023231637800906, Hangzhou, China).

## Conflicts of Interest

The authors declare no conflicts of interest.

## Supporting information


**FIGURE S1.** Upregulation of LYRM2 is observed in HCC cell lines. (A) Protein level of LYRM2 was measured by western blot in 6‐paired HCC tissues and non‐tumour liver tissues. (B, C) qRT‐PCR and western blot were carried out to evaluate the level of LYRM2 expression level in human HCC cell lines (Huh7, Hep3B, MHCC97H, HCCLM3 and MHCC97L) and immortalised human hepatocyte MIHA.

**FIGURE S2.** Establishment of HCC cell lines with LYRM2 overexpression. (A–D) LYRM2 expression vector or control empty vector was transfected into Hep3B and Huh7 cells. LYRM2 overexpression was confirmed by qRT‐PCT and western blot in Hep3B (A, B) and Huh7 (C, D) cells.

**FIGURE S3.** Establishment of HCC cell lines with LYRM2 knockdown. (A–D) LYRM2 shRNA (#1 or #2) or negative control shRNA was transfected into MHCC97H and HCCLM3 cells. LYRM2 knockdown was confirmed by qRT‐PCT and western blot in MHCC97H (A, B) and HCCLM3 (C, D) cells.

**FIGURE S4.** LYRM2 promotes EMT of HCC cells. (A, B) Effects of LYRM2 overexpression on the mRNA level of EMT maker (E‐cadherin, Ncadherin and Vimentin) were evaluated in Hep3B (A) and Huh7 (B) cells by qRT‐PCT. (C, D) Effects of LYRM2 knockdown on the mRNA level of EMT maker (E‐cadherin, N‐cadherin and Vimentin) were evaluated in MHCC97H (C) and HCCLM3 (D) cells by qRT‐PCR.

**FIGURE S5.** The level of LYRM2 is positively correalted with HIF‐1α level in HCC. Correlation analysis for the expression level of LYRM2 and the expression level of HIF‐1α based on the data in TCGA database.

**FIGURE S6.** LYRM2 has no effect on HIF‐2α level in HCC cells. (A) The influence of LYRM2 overexpression on HIF‐2α protein level was determined in Hep3B and Huh7 cells. (B) The influence of LYRM2 knockdown on HIF‐2α protein level was determined in MHCC97H and HCCLM3 cells.

**FIGURE S7.** LYRM2 knockdown reduces HIF‐1α protein level in subcutaneous tumour tissues. IHC staining was performed to demonstrate the effect of LYRM2 knockdown on the level of HIF‐1α, p53, c‐Myc and HIF‐2α protein in xenograft tumours.

**Table S1.**.

## Data Availability

All data in this study are available upon request by contact with the corresponding author.
