# Supplementary figures and images for: LYRM2 Promotes the Growth and Metastasis of Hepatocellular Carcinoma via Enhancing HIF‐1α‐Dependent Glucose Metabolic Reprogramming
Source: J Cell Mol Med. 2024 Dec 11;28(23):e70241. doi: 10.1111/jcmm.70241 (PMC11633053; doi:10.1111/jcmm.70241)

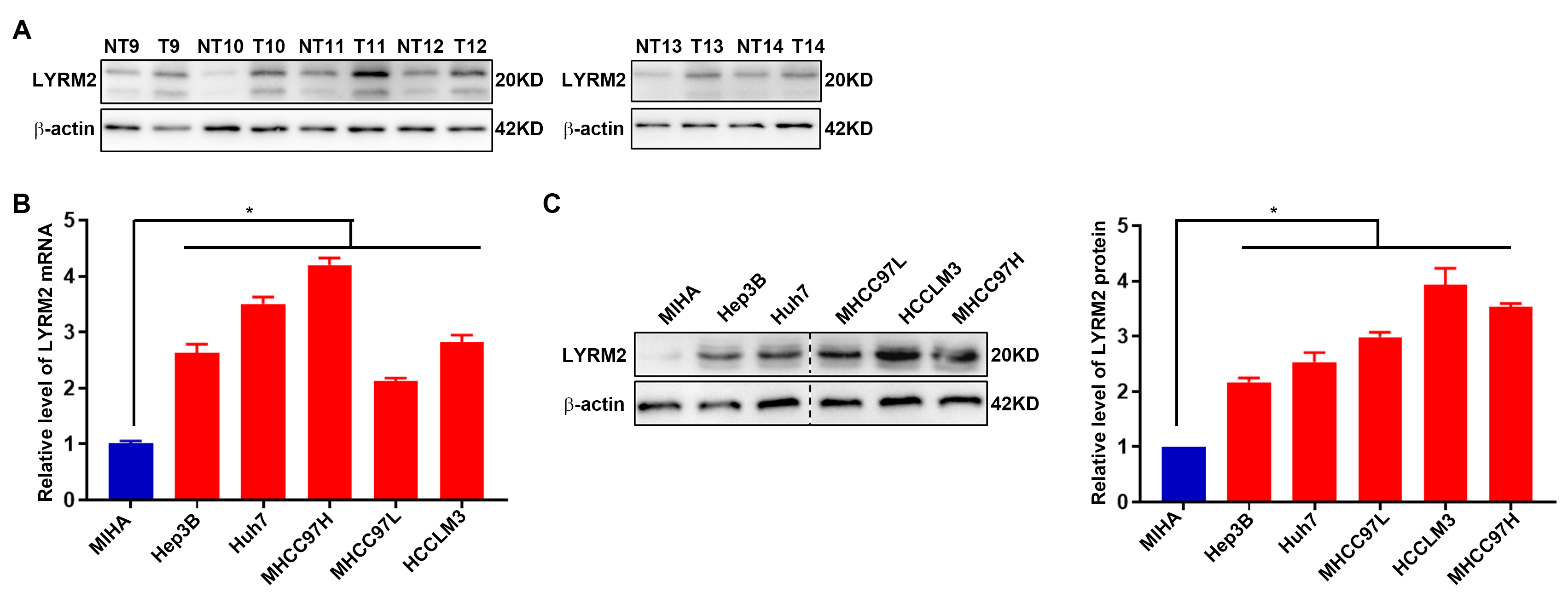

Supplement: Supplementary file 1 — FIGURE S1. Upregulation of LYRM2 is observed in HCC cell lines. (A) Protein level of LYRM2 was measured by western blot in 6‐paired HCC tissues and non‐tumour liver tissues. (B, C) qRT‐PCR and western blot were carried out to evaluate the level of LYRM2 expression level in human HCC cell lines (Huh7, Hep3B, MHCC97H, HCCLM3 and MHCC97L) and immortalised human hepatocyte MIHA. [file JCMM-28-e70241-s004.tif]

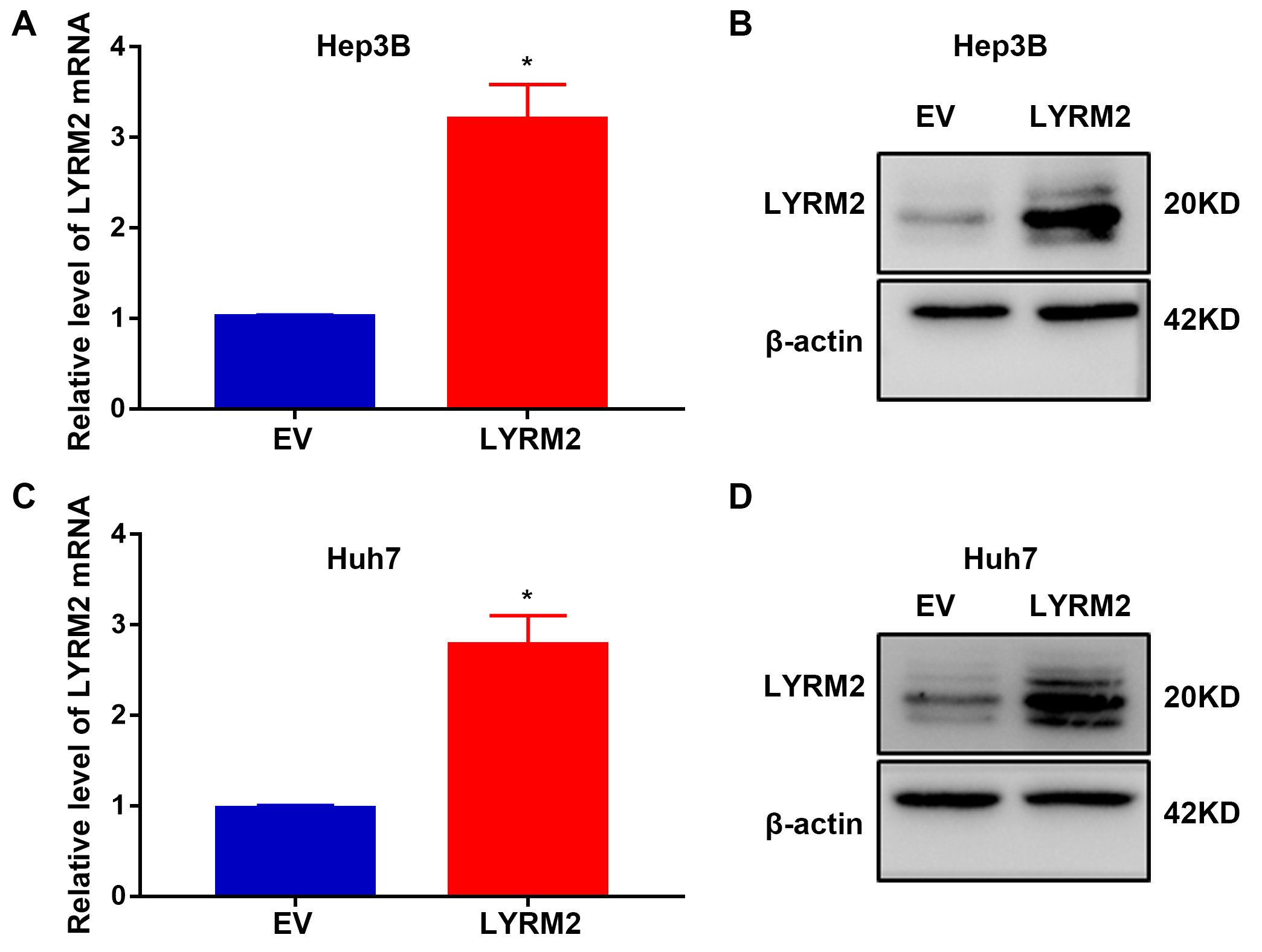

Supplement: Supplementary file 2 — FIGURE S2. Establishment of HCC cell lines with LYRM2 overexpression. (A–D) LYRM2 expression vector or control empty vector was transfected into Hep3B and Huh7 cells. LYRM2 overexpression was confirmed by qRT‐PCT and western blot in Hep3B (A, B) and Huh7 (C, D) cells. [file JCMM-28-e70241-s008.tif]

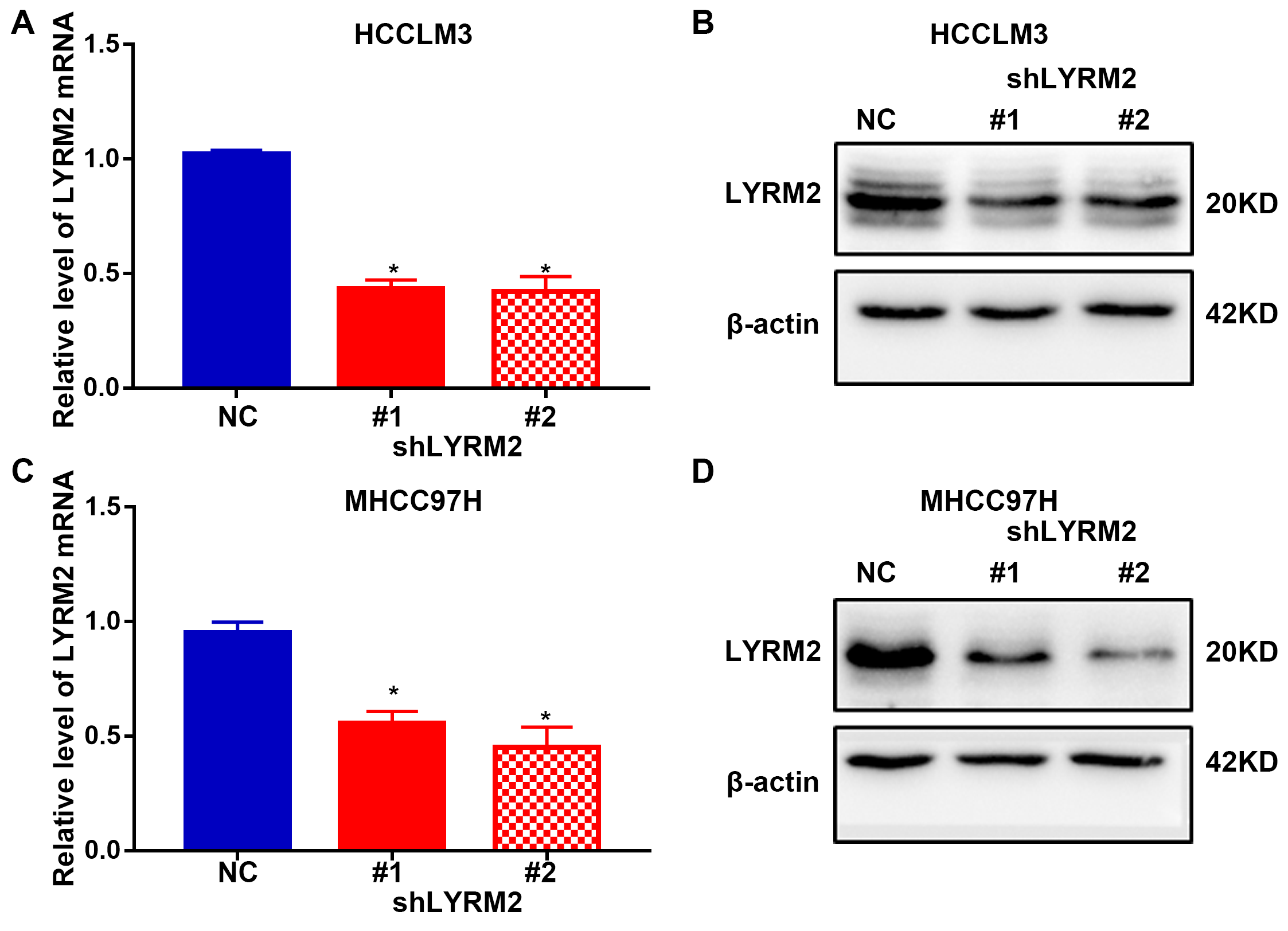

Supplement: Supplementary file 3 — FIGURE S3. Establishment of HCC cell lines with LYRM2 knockdown. (A–D) LYRM2 shRNA (#1 or #2) or negative control shRNA was transfected into MHCC97H and HCCLM3 cells. LYRM2 knockdown was confirmed by qRT‐PCT and western blot in MHCC97H (A, B) and HCCLM3 (C, D) cells. [file JCMM-28-e70241-s001.tif]

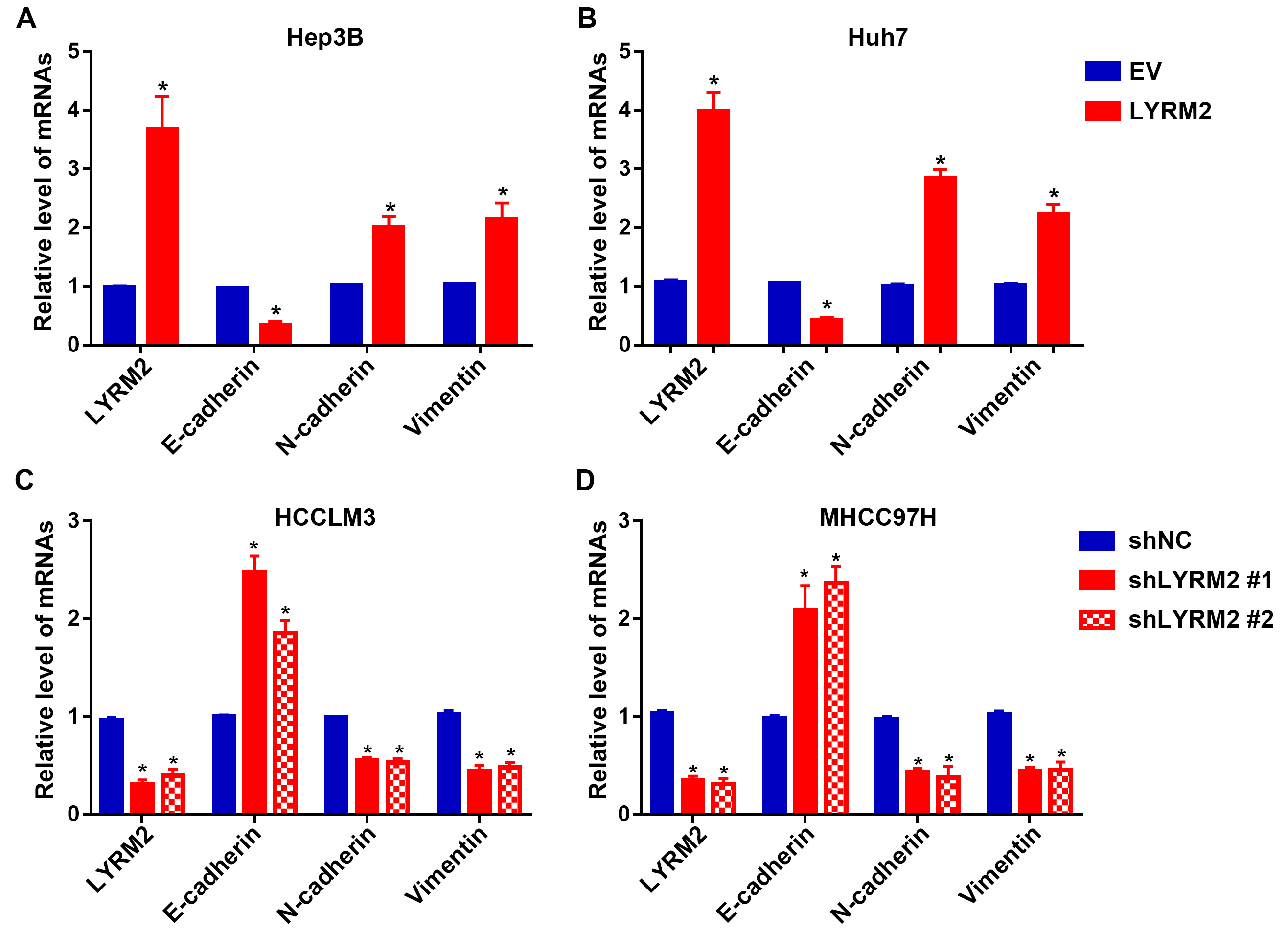

Supplement: Supplementary file 4 — FIGURE S4. LYRM2 promotes EMT of HCC cells. (A, B) Effects of LYRM2 overexpression on the mRNA level of EMT maker (E‐cadherin, Ncadherin and Vimentin) were evaluated in Hep3B (A) and Huh7 (B) cells by qRT‐PCT. (C, D) Effects of LYRM2 knockdown on the mRNA level of EMT maker (E‐cadherin, N‐cadherin and Vimentin) were evaluated in MHCC97H (C) and HCCLM3 (D) cells by qRT‐PCR. [file JCMM-28-e70241-s006.tif]

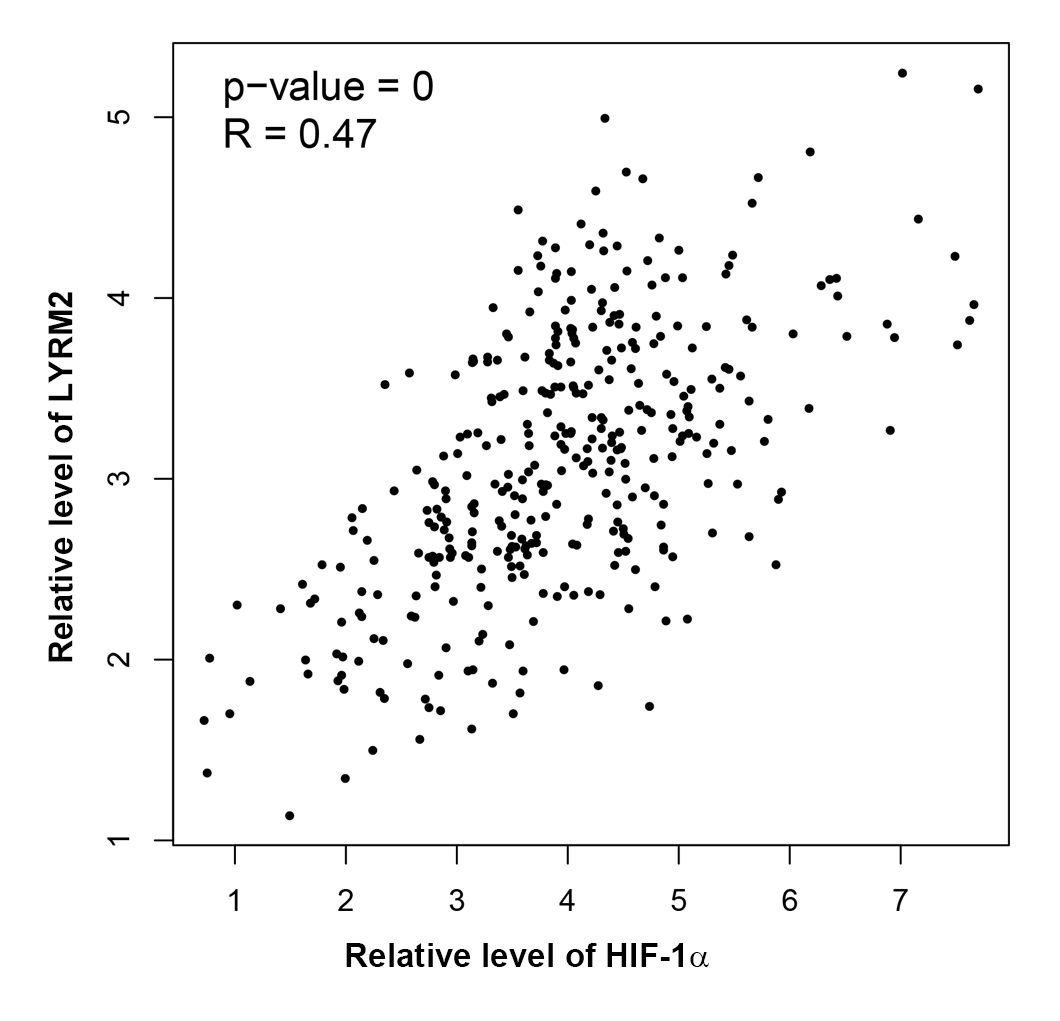

Supplement: Supplementary file 5 — FIGURE S5. The level of LYRM2 is positively correalted with HIF‐1α level in HCC. Correlation analysis for the expression level of LYRM2 and the expression level of HIF‐1α based on the data in TCGA database. [file JCMM-28-e70241-s002.tif]

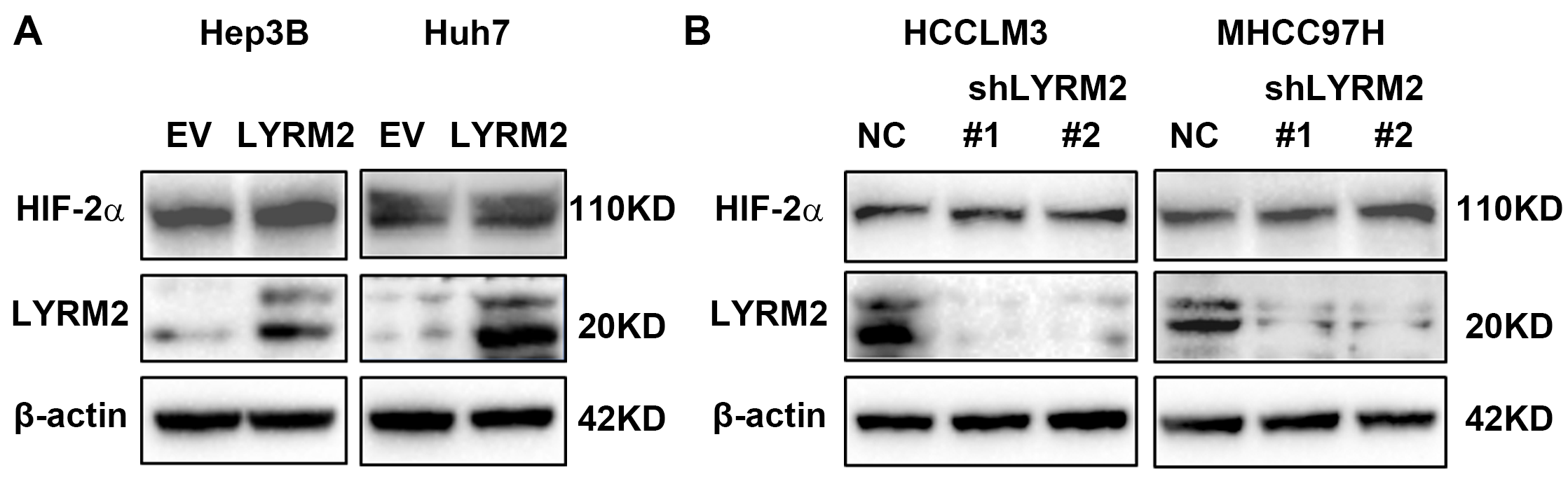

Supplement: Supplementary file 6 — FIGURE S6. LYRM2 has no effect on HIF‐2α level in HCC cells. (A) The influence of LYRM2 overexpression on HIF‐2α protein level was determined in Hep3B and Huh7 cells. (B) The influence of LYRM2 knockdown on HIF‐2α protein level was determined in MHCC97H and HCCLM3 cells. [file JCMM-28-e70241-s003.tif]

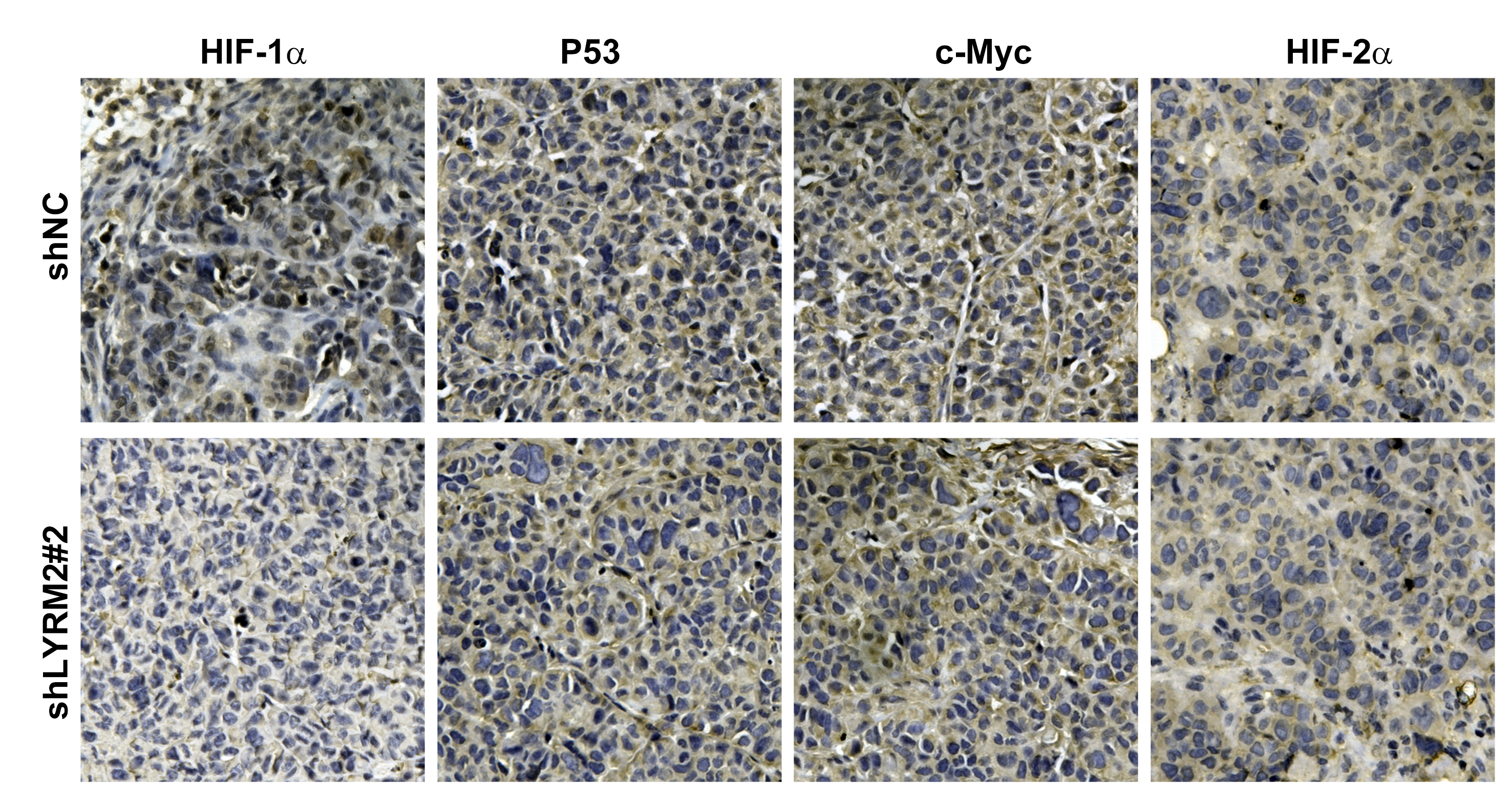

Supplement: Supplementary file 7 — FIGURE S7. LYRM2 knockdown reduces HIF‐1α protein level in subcutaneous tumour tissues. IHC staining was performed to demonstrate the effect of LYRM2 knockdown on the level of HIF‐1α, p53, c‐Myc and HIF‐2α protein in xenograft tumours. [file JCMM-28-e70241-s005.tif]
