# Supplementary material for: LYRM2 Promotes the Growth and Metastasis of Hepatocellular Carcinoma via Enhancing HIF‐1α‐Dependent Glucose Metabolic Reprogramming
Source: J Cell Mol Med. 2024 Dec 11;28(23):e70241. doi: 10.1111/jcmm.70241 (PMC11633053; doi:10.1111/jcmm.70241)
Supplement: Supplementary file 8 — Table S1.. [file JCMM-28-e70241-s007.docx]

Supplementary Table 1. Primer sequences for targeted genes

| Genes | Primer sequences | |
| --- | --- | --- |
| LYRM2 | Forward | 5’-ATGGCTGCTTCCCGCTTAC-3’ |
|  | Reverse | 5’- TTGCCTTCTTACGAACTGCT-3’ |
| E-cadherin | Forward | 5’-CGAGAGCTACACGTTCACGG-3’ |
|  | Reverse | 5’-GGGTGTCGAGGGAAAAATAGG-3’ |
| N-cadherin | Forward | 5’-TCAGGCTGTGGACATAGAAACC-3’ |
|  | Reverse | 5’-GCTGTAAACGACTCTGGCACT-3’ |
| Vimentin | Forward | 5’-CCAAACTTTTCCTCCCTGAACC-3’ |
|  | Reverse | 5’-CGTGATGCTGAGAAGTTTCGTTGA-3’ |
| PGK2 | Forward | 5’-AAACTGGATGTTAGAGGGAAGCG-3' |
|  | Reverse | 5’-GGCCGACCTAGATGACTCATAAG-3' |
| PDK1 | Forward | 5’-CTGTGATACGGATCAGAAACCG-3' |
|  | Reverse | 5’-TCCACCAAACAATAAAGAGTGCT-3' |
| PKM2 | Forward | 5’-ATGTCGAAGCCCCATAGTGAA-3’ |
|  | Reverse | 5’-TGGGTGGTGAATCAATGTCCA-3’ |
| GAPDH | Forward | 5'-AGAAGGCTGGGGCTCATTTG-3' |
|  | Reverse | 5'-AGGGGCCATCCACAGTCTTC-3' |
| LDHA | Forward | 5’-ATGGCAACTCTAAAGGATCAGC-3' |
|  | Reverse | 5’-CCAACCCCAACAACTGTAATCT-3' |
| TPI1 | Forward | 5’- CCCAGGAAGTACACGAGAAG-3’ |
|  | Reverse | 5’-CAGTCACAGAGCCTCCATAAA-3’ |
| PEKL | Forward | 5’-GCTGGGCGGCACTATCATT-3' |
|  | Reverse | 5’-TCAGGTGCGAGTAGGTCCG-3' |
| GPL1 | Forward | 5’-CAGAAGTTGGTCGTGAGGCA-3' |
|  | Reverse | 5’-GCCTTTCACCAGCCAAGCAA-3' |
| GLUT1 | Forward | 5’-CTTTGTGGCCTTCTTTGAAGT-3' |
|  | Reverse | 5’-CCACACAGTTGCTCCACAT-3' |
| HK2 | Forward | 5’-TTGACCAGGAGATTGACATGGG-3' |
|  | Reverse | 5’-CAACCGCATCAGGACCTCA-3' |
| PGAM2 | Forward | 5’-AGAAGCACCCCTACTACAACTC-3' |
|  | Reverse | 5’-TCTGGGGAACAATCTCCTCGT-3' |
| ENO1 | Forward | 5’-CCCAGCTGCCCGATCAGTG-3' |
|  | Reverse | 5’-GATGAGCCCAGTTCGGATCTC-3' |
| ALDOA | Forward | 5'- CGGGAAGAAGGAGAACCTG-3' |
|  | Reverse | 5'- GACCGCTCGGAGTGTACTTT-3' |
| P53 | Forward | 5'- CTGAGGTTGGCTCTGACTGTACCACCATCCTCCTCC-3' |
|  | Reverse | 5'- CTCATTCAGCTCTCGGAACATCTCGAAGCG-3' |
| c-Myc | Forward | 5'- CTTCTCTCC GTCCTCGGATTCT-3' |
|  | Reverse | 5'- GAAGGTGATCC AGACTCTGACCTT-3' |
| HIF-1α | Forward | 5'- CTCCCATACAAGGCAGCAGAAA-3' |
|  | Reverse | 5'- CAAAACAACCAACAGAAACGAAAC-3' |
| β-actin | Forward | 5'- GGGAAATCGTGCGTGACATTAAG-3' |
|  | Reverse | 5'- TGTGTTGGCGTACAGGTCTTTG-3' |
